# Peptides adopt stable omega-loop structures in concentrated sulfuric acid

**DOI:** 10.1073/pnas.2618039123

**Published:** 2026-09-04

**Authors:** Jia Yi Zhang, Aurelio J. Dregni, Janusz J. Petkowski, Sara Seager, Mei Hong

**Affiliations:** ^a^ Department of Chemistry, Massachusetts Institute of Technology, Cambridge, MA 02139; ^b^ Faculty of Environmental Engineering, Wroclaw University of Science and Technology, Wroclaw 50-370, Poland; ^c^JJ Scientific, Warsaw, Mazowieckie 02-792, Poland; ^d^ Department of Earth, Atmospheric and Planetary Sciences, Massachusetts Institute of Technology Cambridge, MA 02139; ^e^ Department of Physics, Massachusetts Institute of Technology, Cambridge, MA 02139; ^f^ Department of Aeronautics and Astronautics, Massachusetts Institute of Technology, Cambridge, MA 02139

**Keywords:** peptides, peptide folding, concentrated sulfuric acid, Venus

## Abstract

The search for life beyond Earth has focused on planets and moons with liquid water, reflecting the assumption that complex biomolecules require aqueous environments to remain stable and functional. Such a view excludes a wide class of planetary settings, including the concentrated sulfuric acid clouds of Venus, where extreme acidity and minimal water are thought to preclude molecular structure despite suitable temperatures. A growing body of evidence shows that a wide range of organic molecules can remain stable in concentrated sulfuric acid, including nucleic acid bases, amino acids, lipid micelles and vesicles, and peptide nucleic acid (PNA), but such stability does not address whether macromolecules can retain folded structures required for function. Using NMR spectroscopy, here we show that three peptides adopt stable, well-defined folded structures in concentrated (98% w/w) sulfuric acid, an extreme solvent environment that has been long assumed to be incompatible with biomolecules. The peptides form compact Ω-loop conformations stabilized by solvent-mediated interactions and intramolecular hydrogen bonding. This finding strongly counters conventional thinking where sulfuric acid would destroy peptide bonds via acid-catalyzed hydrolysis. Here, the near absence of water in 98% (w/w) sulfuric acid means hydrolysis does not occur. The result identifies a regime in which macromolecular structure persists under conditions long considered incompatible with life. The finding expands the range of planetary environments that may support complex chemistry and motivates renewed consideration of chemically diverse exoplanets in the search for signs of life beyond Earth.

## Significance

Concentrated sulfuric acid is widely regarded as destructive to Earth-like biochemistry. Recent studies, however, show that an increasing number of biomolecules, including nucleic acid bases, amino acids, and lipids, remain stable in this solvent. A central question is whether such stability extends to macromolecules that require persistent three-dimensional structures for function. Using NMR spectroscopy, here we show that three peptides adopt stable folded structures in concentrated sulfuric acid. With only trace water present, acid-catalyzed hydrolysis is suppressed. The peptides form compact Ω-loop conformations stabilized by solvent-mediated interactions and intramolecular hydrogen bonding. The persistence of folded peptides in concentrated sulfuric acid expands the range of liquid environments that may support macromolecular structure and function, including Venus clouds and exoplanets.

The peptide bond is one of the most important chemical bonds in biochemistry. It links amino acids into large polymers—proteins—that derive function from their ability to fold into defined three-dimensional structures. For this reason, peptide bonds are a natural starting point when considering the molecular basis of life beyond Earth, as they provide a proven route to large, information-rich, biologically functional polymers.

The diversity of Solar System bodies and exoplanets motivates the investigation of nonaqueous solvents for life (1). One intriguing environment is the cloud layer of Venus, where temperate conditions coexist with droplets of concentrated sulfuric acid (2). In this environment, the most basic conditions for life exist: suitable temperatures for covalent bonds, a liquid medium, and an energy source, the Sun (3). Any life in such conditions would require molecular building blocks that remain stable and functional in a strongly acidic and highly protonating solvent. Yet, select biomolecules—including nucleic acid bases (4, 5), single strand PNA (6), amino acids (7), and some lipids (8)—have recently been shown to be stable in concentrated sulfuric acid, motivating a closer examination of whether more complex, structured polymers, for example peptides, can also persist.

**Fig. 1.**
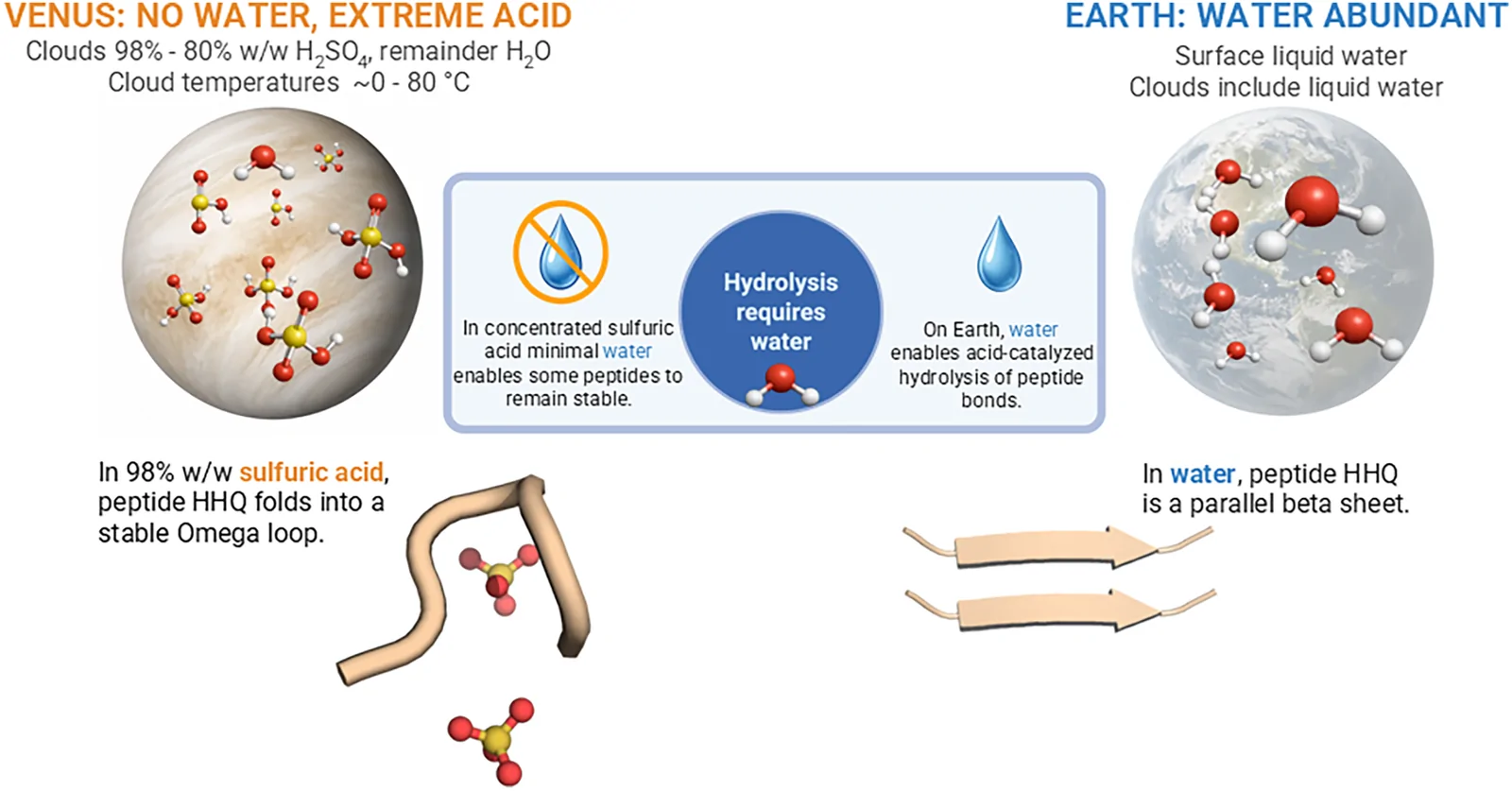
In 98% H_2_SO_4_ the acid-catalyzed hydrolysis of the peptide bond is suppressed, allowing peptides to not only remain stable but also fold into defined three-dimensional structures.

Conventional understanding holds that peptide bonds cannot survive in concentrated sulfuric acid. The peptide bond is susceptible to acid-catalyzed hydrolysis, in which protonation of the carbonyl group in the peptide linkage accelerates bond cleavage (9). Concentrated sulfuric acid, however, is a chemically very different substance from its water-diluted counterpart. At 98% w/w concentrations (hereafter referred to 98%), the amount of remaining water is very low, and sulfuric acid dominates the chemistry of the solvent. Thus, canonical acid-catalyzed hydrolysis may not occur, which would entirely change the chemical behavior of biopolymers (Fig. 1) (6, 10). We are therefore motivated to explore peptide behavior in concentrated sulfuric acid.

## Results

### HHQ Peptide Is Stable in 98% H_2_SO_4_ and Is Well Ordered.

**Fig. 2.**
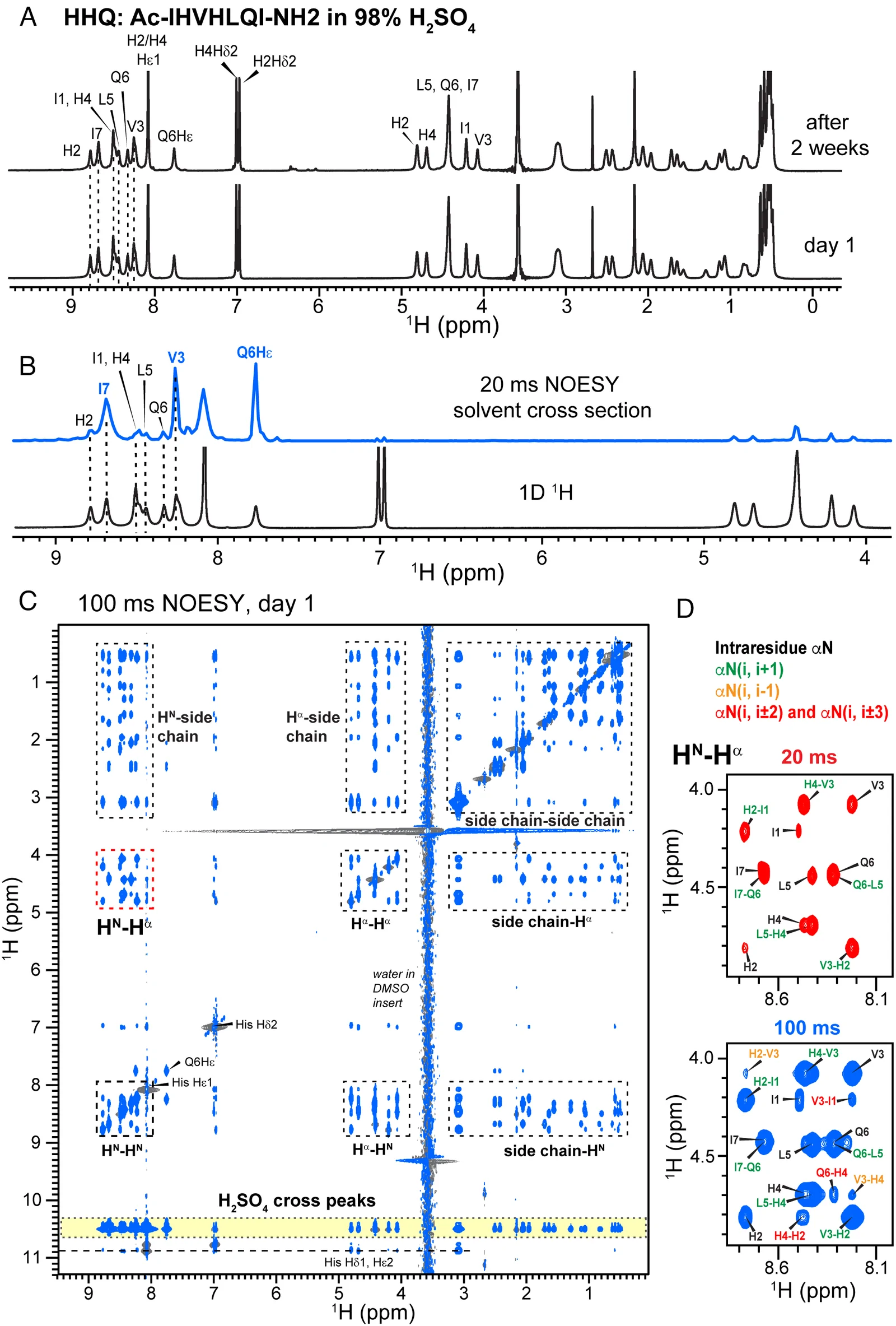
One- and two-dimensional ^1^H NMR spectra of HHQ in 98% H_2_SO_4_ indicate a stable and defined structure. (*A*) ^1^H spectra of the peptide on day 1 and after 2 wk of being in H_2_SO_4_ at room temperature. (*B*) Solvent cross-section of the 2D NOESY spectrum compared to the equilibrium ^1^H spectrum. High solvent crosspeaks with Val3 and Ile7 H^N^ and Gln6 Hε indicate that these protons are in close contact with solvent protons. (*C*) 2D ^1^H-^1^H NOESY spectrum with 100 ms mixing measured on day 1. Strong peptide-solvent crosspeaks are observed at ~10.5 ppm in the ω_1_ dimension (yellow shaded region). (*D*) H^N^-H^α^ fingerprint region of the NOESY spectra at 20 ms and 100 ms mixing. Sequential H^α^- H^N^ crosspeaks have higher intensities than intraresidue H^α^- H^N^ peaks.

We first studied the heptapeptide Ac-IHVHLQI-NH_2_, termed HHQ, which assembles into β-sheet amyloid fibrils in water (Fig. 1) (11). HHQ readily dissolved in the viscous 98% H_2_SO_4_ at a concentration of 20 mg/mL and formed a transparent solution. Proton NMR spectra show well dispersed amide proton (H^N^) and aliphatic proton signals at chemical shifts that are typical for proteins in aqueous solution. The spectra were unchanged for several weeks at ambient temperature, indicating that HHQ is stable in this solvent (Fig. 2*A*). Even after 1 mo, the spectra remained very similar, except for a few weak peaks that appeared in the amino region (6.0 to 6.5 ppm), suggesting a low level of degradation. It is worth noting that high-temperature NMR experiments are prohibitive for these samples due to the high conductivity of the solvent and the resulting strong radiofrequency power levels, which could damage the NMR probe.

**Fig. 3.**
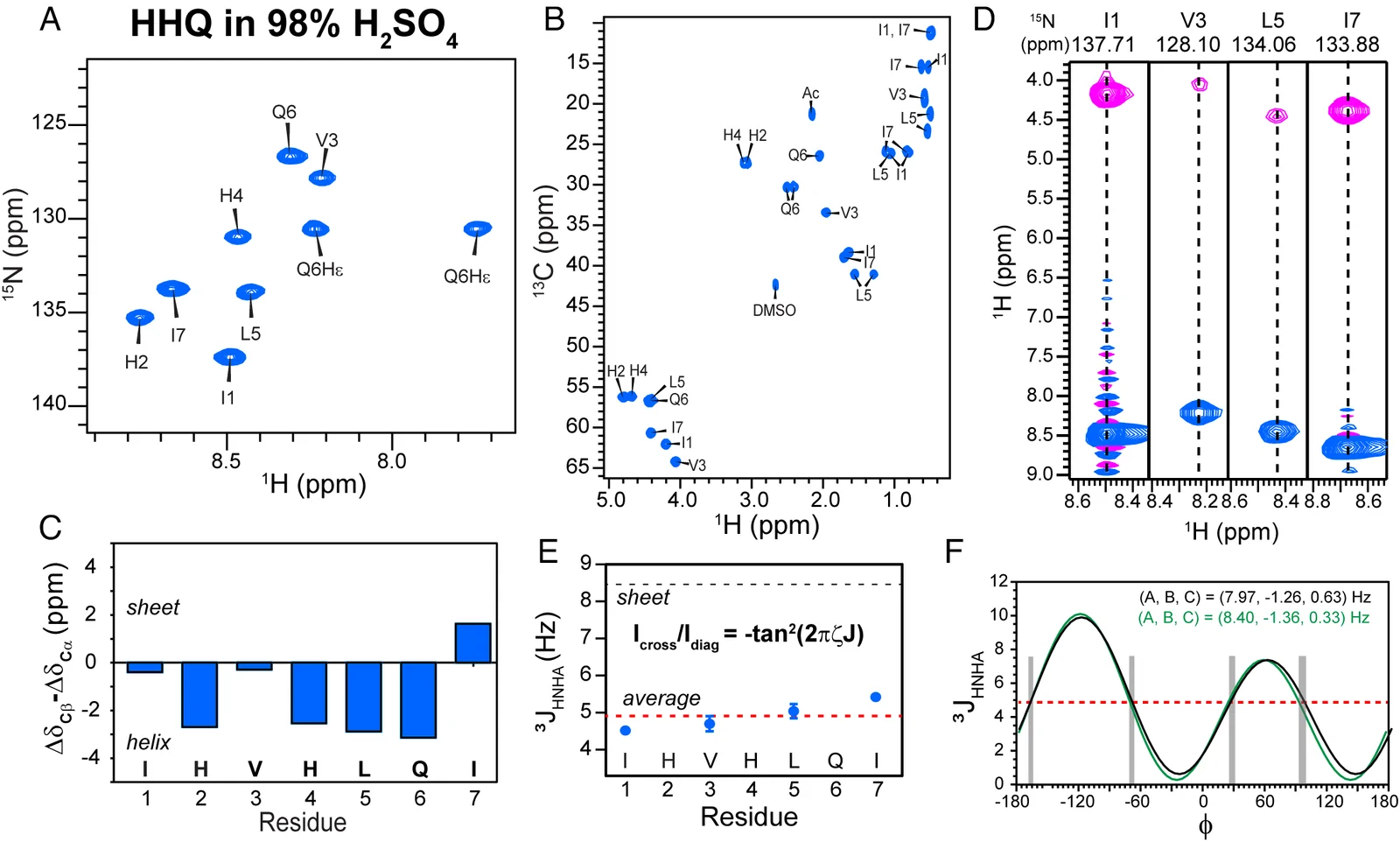
2D and 3D heteronuclear correlation spectra of HHQ in 98% H_2_SO_4_, giving conformation-dependent ^13^C chemical shifts and backbone ϕ torsion angles. (*A*) 2D ^1^H-^15^N HSQC spectrum. (*B*) Aliphatic region of the 2D ^1^H-^13^C HMQC spectrum. (*C*) Cα and Cβ secondary chemical shifts. (*D*) Strips of 3D J_HNHA_ spectrum for labeled residues. Positive intensities for H^N^ diagonal peaks are shown in blue and negative intensities for H^N^-H^α^ crosspeaks are shown in pink. (*E*) ^3^J_HNHA_ couplings calculated from the crosspeak to diagonal peak intensity ratio. (*F*) Karplus curves indicating the ϕ torsion angle ranges that are consistent with the measured ^3^J_HNHA_ couplings of ~5 Hz. Curves were calculated using both rigid-limit (black) and ensemble-averaged (green) Karplus coefficients A, B, C (13).

We assigned ^1^H, ^13^C, and ^15^N chemical shifts of HHQ using two-dimensional (2D) ^1^H-^1^H Nuclear Overhauser Effect spectroscopy (NOESY) (12) (Fig. 2*C* and *SI Appendix*, Fig. S1), ^1^H-^13^C heteronuclear multiple-quantum coherence (HMQC) and ^1^H-^15^N heteronuclear single-quantum coherence (HSQC) (Fig. 3 *A* and *B*) experiments. The spectra show a single set of chemical shifts, consistent with a homogeneous structure. Because concentrated sulfuric acid is 25-fold more viscous than water, this short peptide exhibits strong NOE crosspeaks like a ~20 kDa protein in aqueous solution. Strikingly, the backbone amide protons show strong correlations with solvent protons at 10.5 ppm (Fig. 2*B*). These correlations are stronger for Val3 and Ile7 than for His2, His4, and Gln6, indicating that even though the neighboring H^N’^s are separated by only 4 Å, they have distinct solvent accessibilities. Thus, HHQ adopts a stable conformation in 98% H_2_SO_4_ and interacts strongly with the solvent.

To obtain information about the HHQ conformation in concentrated sulfuric acid, we measured NOESY spectra using mixing times of 20 to 300 ms. Interestingly, the peptide shows stronger sequential H^α^*_i_*-H^N^*_i_*_+1_ correlations than intraresidue H^α^*_i_*-H^N^*_i_* correlations at short mixing times (Fig. 2*D* and *SI Appendix*, Fig. S1), which is typically diagnostic of extended β-strands. However, β-strands have weak sequential H^N^-H^N^ correlations and no medium and long-range correlations (12), whereas HHQ exhibits many sequential and medium-range crosspeaks (*SI Appendix*, Fig. S1). Moreover, Cα and Cβ chemical shifts indicate a helix-like conformation for the peptide in 98% sulfuric acid (Fig. 3*C*), in contrast to the weakly β-strand chemical shifts found in water (*SI Appendix*, Fig. S2). To further clarify the backbone conformation, we measured three-bond H^N^-H^α^ J couplings (^3^J_HNHA_) (14), which depend on the ϕ torsion angle: ^3^J_HNHA_ values of ~4 Hz are indicative of α-helical ϕ angles of about −60° whereas larger values of ~9 Hz indicate β-strand ϕ angles of about −130°. We labeled Ile1, Val3, Leu5, and Ile7 and measured their J couplings using the 3D HNHA experiment (Fig. 3 *D* and *E*). The relative intensities of H^N^-H^N^ and H^N^-H^α^ crosspeaks yielded ^3^J_HNHA_ values of 4.5 to 5.5 Hz for all labeled residues. These values correspond to ϕ angles of about −70°, −165°, 30°, and 100°, whether rigid-limit or ensemble-averaged Karplus coefficients are used (Fig. 3*F*) (13), thus excluding the β-strand conformation. Together, these sequential H^α^*_i_*-H^N^*_i_*_+1_ and H^N^*_i_*-H^N^*_i_*_+1_ crosspeaks, helix-like ^13^C chemical shifts, and small ^3^J_HNHA_ couplings, indicate that HHQ in concentrated sulfuric acid is neither a canonical helix nor a canonical strand.

To investigate whether these unusual properties of HHQ result from internal flexibility of the peptide despite the high viscosity of concentrated sulfuric acid, we measured ^15^N longitudinal (R_1_) and transverse (R_2_) relaxation rates and ^15^N-^1^H heteronuclear NOEs (15, 16) for the four ^15^N-labeled residues, Ile1, Val3, Leu5, and Ile7 (*SI Appendix*, Fig. S3). Residues Val3 and Leu5 in the middle of the peptide show moderate R_1_, high R_2_, and high NOE values of ~0.8 s^−1^, 30 s^−1^, and ~0.80, whereas the two terminal residues, Ile1 and Ile7, show lower R_2_ values. Analysis of these relaxation rates using the model-free Lipari-Szabo formalism (17) yielded high-order parameters of ~0.85 for the middle portion of the peptide, indicating that HHQ adopts a well-ordered structure rather than a conformationally averaged ensemble (*SI Appendix*, Table S1). Ile1 and Ile7 have lower-order parameters of 0.36 and 0.62, consistent with moderate flexibility of the termini. The rotational correlation time of the peptide is 19.3 ns whereas the internal correlation times τ_e_ are 1 to 2 ns, indicating that the viscous concentrated sulfuric acid dramatically reduced both rotational and internal peptide dynamics relative to what would occur in water.

### HHQ Forms an Ω-Shaped Loop Stabilized by Interactions with Sulfuric Acid Molecules.

The well-ordered nature of HHQ in 98% sulfuric acid allowed us to determine its structure using NOESY crosspeak intensities following established protocols (12). Intraresidue and sequential H^α^-H^N^ crosspeaks increased their intensities from 20 to 100 ms with approximately linear slopes (*SI Appendix*, Fig. S4*A*), indicating that these crosspeaks reflect direct contacts. We assigned peaks first observed at 20 ms, 50 ms, 100 ms, and 300 ms to distance upper bounds of 4.5 Å, 5.5 Å, 6.0 Å, and 6.5 Å, respectively (*SI Appendix*, Table S2). These distance upper bounds are conservatively long to account for potential relayed transfer at long mixing times. These restraints were combined with ^3^J_HNHA_ values for structure calculation (Table 1 and *SI Appendix*, Table S3).

**Table 1.** NMR restraints and structure calculation statistics of HHQ, HHQ13, and K7 peptides in 98% H_2_SO_4_

NMR distance and angular constraints	HHQ	HHQ13	K7
Distance restraints			
Total NOE	162	277	145
Intraresidue (unambiguous)	60	107	60
Interresidue (unambiguous)			
Sequential (|i − j| = 1)	67	93	39
Medium range (2 ≤ |i − j| ≤ 3)	34	33	13
Long range (|i − j| ≥ 4)	1	4	2
Ambiguous	0	40	31
J-coupling restraints			
^3^J_HNHA_	4	4	6
Structure statistics			
Violations (mean ± SD)			
Distance restraints (Å)	0.043 ± 0.003	0.040 ± 0.004	0.066 ± 0.005
J-coupling restraints (Hz)	0.051 ± 0.005	0.029 ± 0.011	0.058 ± 0.012
Max distance-restraint violation (Å)	0.378	0.5386	0.494
Max J-coupling violation (Hz)	0	0	0
Deviations from idealized geometry			
Bond lengths (Å)	0.006 ± 0.000	0.005 ± 0.000	0.007 ± 0.001
Bond angles (°)	0.675 ± 0.035	0.589 ± 0.033	1.001 ± 0.077
Impropers (°)	0.748 ± 0.036	0.363 ± 0.064	0.864 ± 0.141
Average pairwise rmsd (Å)			
Heavy	0.14 ± 0.17	2.65 ± 0.70	0.81 ± 0.61
Backbone	0.14 ± 0.18	2.07 ± 0.57	0.52 ± 0.40

Pairwise rmsd was calculated as the average rmsd between the lowest energy structure and the other 19 structures in the lowest-energy ensemble.

**Fig. 4.**
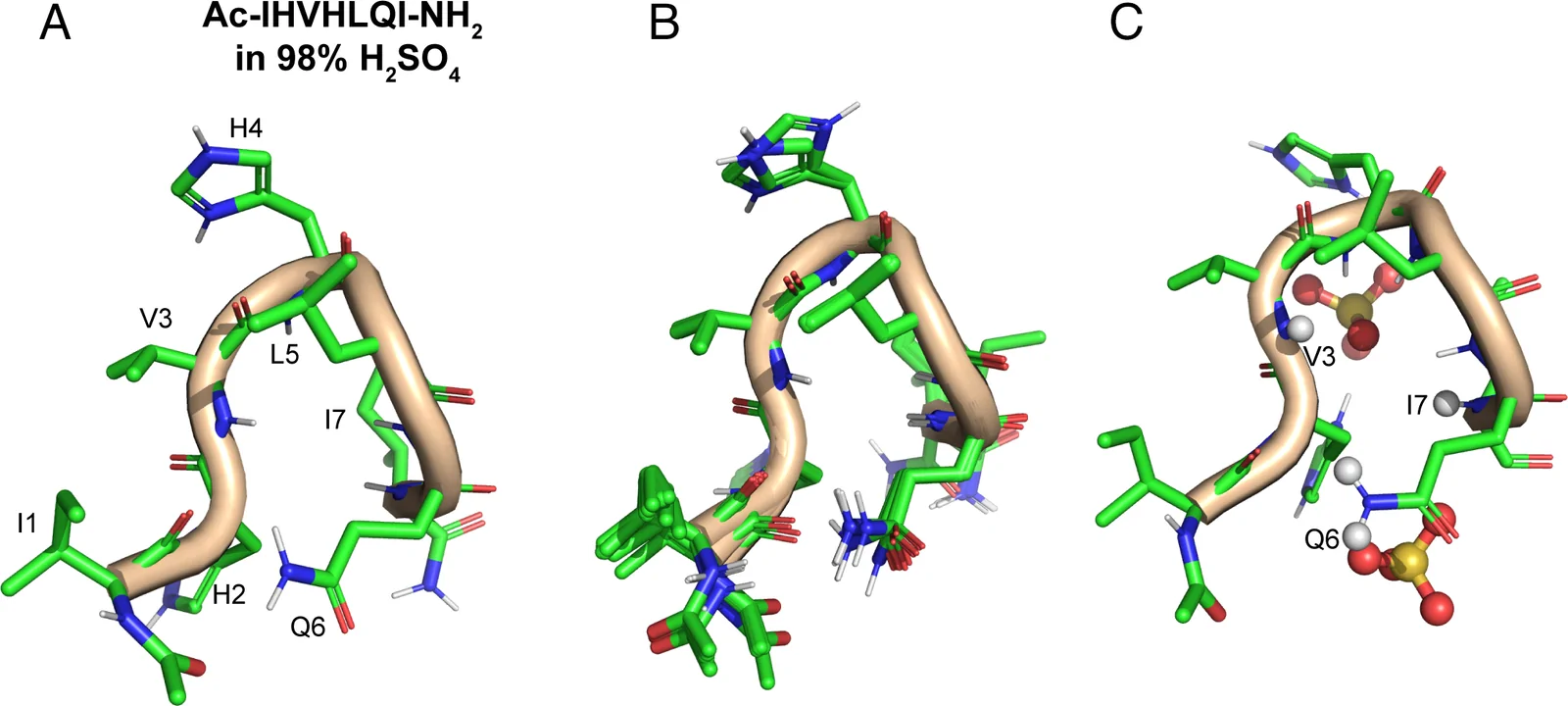
Structural models of HHQ in 98% H_2_SO_4_. (*A*) The lowest-energy structural model shows an Ω loop with short sequential H^α^*_i_*-H^N^*_i_*_+1_ distances. (*B*) Lowest-energy ensemble of 10 structures. (*C*) HHQ with two docked H_2_SO_4_ molecules. The solvent oxygen atoms form hydrogen bonds with multiple peptide NH groups, scaffolding the Ω loop. Val3 and Ile7 H^N^ atoms and Q6 sidechain H^N^ atoms, which show strong solvent NOEs, are shown as spheres.

Remarkably, the lowest-energy structure of HHQ in 98% sulfuric acid is an omega (Ω)-shaped loop (Fig. 4*A*), constrained by the large number of NOEs (*SI Appendix*, Fig. S5*A*). Val3, Gln6, and Ile7 amide protons point to the center of the loop whereas most sidechains point outward toward the solvent. The interior of the loop is spacious, with a void volume of about 162 Å^3^ out of a total peptide volume of 1,001 Å^3^ (18). Intramolecular hydrogen bonds are found between Gln6 sidechain NH_2_ and C═O of the N-terminal acetyl and Ile1, with O^…^H distances of 2.8 Å and 2.3 Å, respectively. The peptide is well ordered, with a heavy-atom rmsd of 0.14 Å for the 20 lowest-energy structure models (Fig. 4*B*), consistent with the measured high-order parameter values. Both His2 and His4 are cationic, as evidenced by the imidazole N^ε2^ and N^δ1^ chemical shifts (169.0 and 169.9 ppm) and C^δ2^ chemical shift (120.5 ppm) (19, 20). When the longest-mixing time (300 ms) distance restraints were excluded from structure calculation, we obtained the same omega-shaped fold (*SI Appendix*, Fig. S5*D*), indicating that the conformation is insensitive to the potential presence of relayed transfer at 300 ms. This likely reflects the low proton density of the peptide, which inhibits spin diffusion, and the conservative distance upper bounds used in structure calculation.

The strong solvent crosspeaks of Val3 H^N^, Ile7 H^N^, and Gln6 sidechain H^ε^ in the NOESY spectra (Fig. 2*B*) suggest that specific solvent locations with high occupancy may exist in close proximity to the peptide. To clarify the nature of this peptide–solvent interaction, we measured a 2D ^1^H-^1^H ROESY spectrum (*SI Appendix*, Fig. S4*B*). With 10 ms mixing, the spectrum shows solvent-H^N^ crosspeaks with the same sign as the diagonal H^N^-H^N^ crosspeaks, indicating that chemical exchange dominates over dipolar coupling for peptide-solvent magnetization transfer (21, 22). To identify plausible solvent positions that are consistent with these crosspeaks and that are compatible with the peptide structure, we used the HADDOCK software to dock H_2_SO_4_ molecules into the HHQ structure (23), specifying Val3, Gln6, and Ile7 as active residues. Simulations using one H_2_SO_4_ molecule did not satisfy all observed solvent crosspeaks whereas simulations using three H_2_SO_4_ molecules led to weak binding energies due to the lack of stabilizing interactions for one of the three molecules. Docking two H_2_SO_4_ molecules yielded the most stable structure (Fig. 4*C* and *SI Appendix*, Table S4). One H_2_SO_4_ molecule lies at the top of the Ω-loop, with oxygen atoms within 1.9 to 4.0 Å of the Val3, His4, Leu5, and Ile7 amide protons and within 2.2 Å of the two imidazolium H^δ1^ protons. The second solvent molecule binds between the N and C termini, interacting with the backbone amides of His2 and Ile7, His2 sidechain H^ε2^, and Gln6 sidechain H^ε^. Given the dynamic nature of the solvent, these docking models are not intended to represent atomic-resolution sites of the nearest solvent molecules. Instead, they indicate that preferred solvent locations with high occupancy exist near the peptide, and that two simultaneous solvent molecules are sufficient to explain the observed site-specific amide-solvent crosspeaks without affecting the peptide structure.

### Omega-Loop Structures are Also Adopted by Other Peptides in 98% Sulfuric Acid.

To investigate whether the Ω-loop structure is more general for peptides in concentrated sulfuric acid, we designed a longer peptide, Ac-IHAHLQAKLHVQI-NH_2_ (HHQ13), which extends the HHQ sequence by six residues and replaces Val3 and Ile7 with Ala. Like HHQ, HHQ13 is soluble and stable in 98% sulfuric acid for many weeks at room temperature, and 2D NOESY spectra show stronger sequential H^α^-H^N^ correlations than intraresidue correlations (*SI Appendix*, Fig. S6). The Cα and Cβ chemical shifts are also consistent with a helix-like conformation (*SI Appendix*, Fig. S7 *B* and *C*). 3D HNHA spectrum resolved Ile1, Ala3, Val11, and Ile13 signals, yielding ^3^J_HNHA_ values of 4.1 – 6.0 Hz (*SI Appendix*, Fig. S7 *A*, *D*, and *E*).

**Fig. 5.**
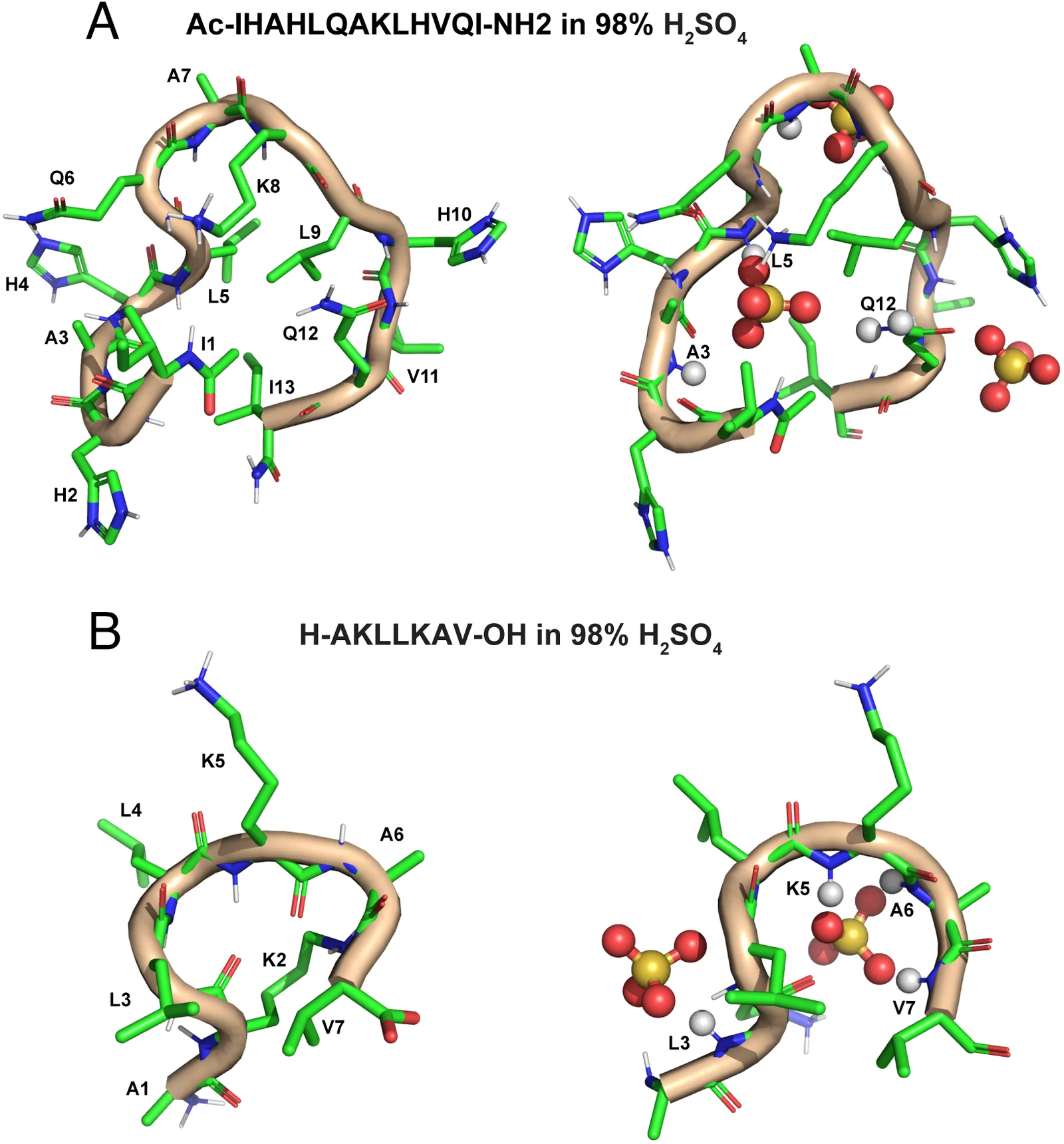
Structural models of HHQ13 and K7 in 98% H_2_SO_4_. (*A*) Lowest-energy model of HHQ13 (*Left*) and the best docking result (*Right*), with three H_2_SO_4_ molecules in close contact with H^N^ atoms of Ala3, Ala7, and Gln12. (*B*) Lowest-energy model of K7 (*Left*) and its best docking result (*Right*), with two H_2_SO_4_ molecules in close contact with H^N^ atoms of Lys2, Lys5, Ala6, and Val7.

Applying the same NOESY analysis as used for HHQ, we found that the lowest-energy structural models of HHQ13 showed longer sequential H^α^-H^N^ distances than intraresidue crosspeaks for many residues, contradicting experimental data. We thus calibrated the distances of the 20 sequential and intraresidue H^α^-H^N^ peaks in the 20 ms NOESY spectrum by comparing their intensities with the Gln12 H^γ1^-H^γ2^ peak, which corresponds to a fixed distance of 1.8 Å (24). The 20 ms NOESY spectrum was chosen for this quantification because the H^α^-H^N^ crosspeak intensities are in the initial buildup regime. With this quantification and based on 277 NOEs and 4 ^3^J_HNHA_ couplings, we obtained a lowest-energy HHQ13 structure model that is in good agreement with experimental data. The structure contains two Ω-loops (Fig. 5*A*): the first spans residues Ile1 to Leu5 whereas the second encompasses Leu5 to Leu9. The backbone of the last four residues makes a bend, bringing the C-terminus close to the N terminus. Like its parent peptide, HHQ13 maintains its omega fold when distance restraints from the 300 ms NOESY spectra are excluded from structure calculation (*SI Appendix*, Fig. S5*E*). The double-omega conformation sequesters multiple sidechains in the interior, including Leu9, Gln12, and Ile13. Numerous polar interactions between amide and carbonyl groups, such as between His2 and the N-terminal acetyl, and between Leu5 and Ala3, stabilize the structure. Similar to HHQ, HHQ13 shows strong solvent crosspeaks with specific amide protons, including Ala3, Ala7, and Gln12 (*SI Appendix*, Fig. S6*B*). Docking simulations indicate that these interactions are satisfied by including three H_2_SO_4_ molecules in the structure model (Fig. 5*A*). One H_2_SO_4_ is sequestered by the segment from Ile1 to Leu5, a second molecule binds between Leu5 and His10, whereas the third solvent molecule lies between His10 and Gln12 sidechains, stabilized by numerous polar interactions.

Finally, we investigated a third peptide, K7 (H-AKLLKAV-OH), which has a distinct amino acid sequence from the two histidine-containing peptides and has unmodified N and C termini. The K7 stability in concentrated sulfuric acid differs from HHQ and HHQ13. Its NMR spectra slowly changed over several weeks, with distinct chemical shifts between the initial and equilibrated states (*SI Appendix*, Figs. S8 and S9). However, all sequential H^α^-H^N^ crosspeaks are present, thus the slow spectral change does not reflect solvolysis. Instead, it most likely results from elimination reaction of the C-terminal Val7, as this residue exhibits only two ^1^H-^13^C correlations, one of which has ^1^H and ^13^C chemical shifts of 1.34 and 27.39 ppm, which are consistent with an allylic methyl group (C═C─CH_3_). In comparison, in water, K7 is stable and shows weakly β-strand chemical shifts (*SI Appendix*, Fig. S10). In concentrated sulfuric acid, most ^3^J_HNHA_ values are below 5 Hz, except for Val7 (*SI Appendix*, Fig. S9). Interestingly, the solvent crosspeak pattern for K7 is similar to its 1D ^1^H spectrum (*SI Appendix*, Fig. S8*B*), indicating that the amide protons in this peptide are more solvent-exposed than those of HHQ and HHQ13. ^15^N relaxation data show order parameters of 0.60 to 0.76 for most residues except for the termini (*SI Appendix*, Fig. S3 *H*–*K* and Table S5), indicating that K7 is more flexible than HHQ in concentrated sulfuric acid; nevertheless, these order parameter values rule out a random coil.

Despite the distinct amino acid sequence, remarkably, the initial state of K7 exhibits the same Ω-loop fold as HHQ and HHQ13 (Fig. 5*B*). The top of the loop is occupied by Lys5 whereas the interior of the loop is stabilized by close contact between the Lys2 carbonyl and the Lys5 amide proton. Similar to the two His-containing peptides, the N and C termini are in close proximity, with Val7 and Leu3 methyl groups within about 5 Å of each other. Docking simulations found two bound H_2_SO_4_ molecules, one surrounded by residues Ala5 to Val7 and the second located in the bend formed by residues Ala1 to Leu4 (Fig. 5*B*).

## Discussion

These results conclusively demonstrate that peptides not only remain stable in concentrated sulfuric acid but also fold into defined three-dimensional structures. NMR spectroscopy is ideally suited for studying peptides in concentrated sulfuric acid, because site-specific measurements of chemical shifts, scalar couplings, and polarization transfer are unaffected by the solvent. By contrast, circular dichroism yielded only featureless, nondiagnostic spectra due to the noncanonical peptide folds. Among the three peptides, HHQ and HHQ13 manifest a single set of chemical shifts and are stable for many weeks, whereas K7 exhibits a predominant set of chemical shifts. ^15^N relaxation data show backbone N-H order parameters of ~0.85 for HHQ and 0.60 to 0.76 for K7, indicating that the viscous solvent restricts the internal dynamics of these peptides in addition to slowing their rotational diffusion. The structural stability of peptides in concentrated sulfuric acid, while initially surprising, are actually understandable: acid hydrolysis of peptide bonds requires water molecules (9), which are nearly completely absent in 98% sulfuric acid.

The three peptides studied here have distinct amino acid sequences, and contain both hydrophobic methyl-rich amino acids such as Val, Ile, and Leu and polar and charged amino acids such as Gln, His, and Lys. The fact that these unrelated sequences all adopt Ω-loop–rich conformations strongly suggests that H_2_SO_4_ plays a critical role in scaffolding the peptide backbone into this conformation. This conclusion is further supported by the fact that HHQ self-assembles into β-sheet amyloid fibrils in water even at low concentration (0.2 mM) (11, 25) whereas in 98% H_2_SO_4_ at a much higher concentration (22 mM), HHQ resists aggregation and instead adopts the omega-loop structure with site-specific solvent interactions.

The Ω-loop structure is distinct from α-helical and β-sheet structures of proteins in aqueous solution, and populates regions of the Ramachandran diagram with positive ψ torsion angles and a distribution of ϕ angles (*SI Appendix*, Fig. S11). These conformations are approximately complementary in shape with the solvent molecules. Docking simulations suggest that numerous N-H^…^O═S hydrogen bonds can exist between the peptide and the solvent, and each H_2_SO_4_ molecule can interact with at least four peptide H^N^ groups with H^…^O distances of 1.7 to 4.0 Å, allowing the solvent to scaffold the peptide structure. The Ω-loop fold may be further fortified by intramolecular hydrogen bonding, which is unexpected in this strongly protonating but water-scarce solvent. Conventional understanding holds that concentrated sulfuric acid should outcompete intramolecular hydrogen bonding, preventing the formation of stable three-dimensional structures. Instead, we observed intramolecular N-H^…^O═C hydrogen bonds between Lys5 H^N^ and Lys2 carbonyl in K7 and between the Gln6 sidechain and N-terminal acetyl in HHQ, the latter driving the close approach of the two termini to complete the omega loop. We did not observe such intramolecular hydrogen bonds between the two termini in HHQ13, which may explain the less compact fold of this peptide. Instead, HHQ13 exhibits intramolecular hydrogen bonds between H^N^ of residue *i* and carbonyl of residue *i*-2, which may enforce the Ω-loop fold.

In aqueous solution, omega loops are present in globular proteins as elements that connect regular secondary structures (26–28). These aqueous omega loops are rich in Gly, Pro, and small hydrophilic residues such as Asn, Asp, and Ser, whereas hydrophobic residues are rare. They are preferentially found on protein surfaces and are thought to mediate molecular recognition and protein folding. In comparison, the omega loops determined in this study in concentrated sulfuric acid are autonomous structures unconnected to α-helical or β-strand structures, and are adopted by sequences rich in hydrophobic residues while devoid of Gly and Pro. Therefore, the structural origin of the omega loops in concentrated sulfuric acid is distinct from that in water. Polypeptide chains must be able to sample all sterically allowed (ϕ, ψ) torsion angles as dictated by the combined effects of the amino acid sequence and the environment. Therefore, it is not surprising that a similar backbone conformation can emerge under vastly different environmental conditions. Whether the omega-loop conformation in concentrated sulfuric acids remains abundant in longer protein sequences and harbors similar functions as their aqueous counterparts remains to be seen.

The planetary implications for folded polypeptides in 98% concentrated sulfuric acid are profound. Liquid concentrated sulfuric acid may be a common exoplanetary solvent (29). Not only does it exist in the Venusian clouds but the range of exoplanet conditions implies a wide range of possibilities for its existence (30–32). Moreover, because stable secondary structure implies function, the stable, folded peptides serve as an illustration that complex biochemical machinery is in principle possible in concentrated sulfuric acid. We emphasize that we did not design the peptides to specifically fold in concentrated sulfuric acid; for ease of interpretation we avoided amino acids that are modified in sulfuric acid (e.g., sulfated and sulfonated) and omitted tryptophan which on its own is unstable in concentrated sulfuric acid (7).

In summary, we have shown three peptides that adopt stable, folded structures in concentrated sulfuric acid. These results fundamentally challenge the assumption that folded peptides cannot exist stably in concentrated sulfuric acid (33). Analogous to water, the observed peptide structures in 98% sulfuric acid are maintained by a cooperative network of hydrogen bonding and peptide–solvent interactions. The resulting structures resemble an omega shape, which differs from the structures of these peptides in water. Future experiments will elucidate the more complete amino acid code of protein structures in this fascinating solvent.

## Materials and Methods

### Preparation of Peptide Samples for NMR Experiments.

Three peptides were used in this study: HHQ (Ac-IHVHLQI-NH_2_), HHQ13 (Ac-IHAHLQAKLHVQI-NH_2_), and K7 (H-AKLLKAV-OH). The peptides were synthesized by Thermo Scientific with ≥95% purity. NMR samples were prepared by dissolving 4 to 10 mg of the peptides in 300 to 400 μL of H_2_SO_4_ (98 wt.% in H_2_O) in glass vials with gentle vortexing. The final concentrations were 20 mg/mL for HHQ, 12 mg/mL for HHQ13 and 10.5 mg/mL for K7. We added 0.25 to 0.5 mg of dimethyl sulfone (MSM) as an internal chemical shift calibration standard (34). The peptide solution was transferred to 5 mm NMR tubes containing a coaxial insert that holds d_6_-DMSO for deuterium lock. The NMR tubes were capped, sealed with Parafilm, and stored at ambient temperature.

### Solution NMR Experiments.

NMR spectra were measured on a Bruker Avance 800 MHz solution NMR spectrometer equipped with a room-temperature 5 mm TXI ^1^H/^13^C/^15^N probe with Z gradient capabilities. ^1^H chemical shifts were referenced internally to MSM at 2.77 ppm on the TMS scale (34), while ^13^C and ^15^N chemical shifts were referenced indirectly through the ratio of the gyromagnetic ratios to the DSS scale for ^13^C and liquid ammonia scale for ^15^N. A sample temperature of 293 K was used for all the samples.

^1^H, ^15^N, and ^13^C chemical shifts were assigned using 2D correlation spectra, including ^1^H-^1^H NOESY (8,192 [F1] × 1,024 [F2] complex data points), ^1^H-^1^H ROESY (8,192 [F1] × 1,024 [F2] complex data points), ^1^H-^13^C HMQC (4,096 [F1] × 512 [F2] complex data points), and ^1^H-^15^N HSQC (3,072 [F1] × 1,024 [F2] complex data points). These 2D spectra were measured with 8 to 64 scans per increment and 2 to 2.5 s recycle delay, resulting in 6 to 24 h of measurement time for each experiment. ^1^H-^1^H ROESY spectrum on HHQ was measured using a 10 ms spin-locking pulse with a radiofrequency field strength of 5 kHz and with the carrier frequency set at the solvent peak of 10.58 ppm. Three-bond ^3^J_HNHA_ couplings were measured using the 3D HNHA experiment (4,096 [F1] × 192 [F2] × 30 [F3] complex data points), with 16 scans per increment and 1.5 s recycle delay.

^15^N relaxation NMR experiments were conducted on ^15^N-labeled HHQ and K7 in 98% H_2_SO_4_ using standard Bruker pulse sequences with sensitivity enhancement and gradient selection. A 5 s recycle delay was used in all experiments. Steady-state ^1^H-^15^N NOE values were measured as a pseudo-3D ^1^H-^15^N HSQC spectrum (3,072 [F1] × 32 [F2]) with and without 120° ^1^H presaturation pulses during the recycle delay. ^15^N longitudinal relaxation times (T_1_) were measured as a pseudo-3D ^1^H-^15^N HSQC spectrum (3,072 [F1] × 32 [F2]) with 16 delays from 0 to 1.5 s in 0.1 s increments. ^15^N transverse relaxation times (T_2_) were measured as a pseudo-3D ^1^H-^15^N HSQC spectrum (3,072 [F1] × 32 [F2]) with 16 delays from 0 to 0.14 s in 0.0088 s increments. Each 8.8 ms block involved eight ^15^N 180° pulses of 200 µs each at 1.1 ms intervals.

### Solution NMR Data Analysis.

For ^15^N relaxation data of HHQ and K7, ^1^H-^15^N NOE values were measured as the ratios of peak heights in spectra obtained with and without ^1^H presaturation. The uncertainty of NOE values was propagated from peak signal-to-noise ratios. ^15^N R_1_ and R_2_ values were obtained from best-fit single-exponential decays to the experimental data. The uncertainties from spectral noise were 0.01% of the relaxation rates and are thus not shown in plots. Order parameters S^2^, the global rotational correlation time τ_m_ and site-specific internal correlation time τ_e_ were calculated from the ^15^N R_1_, R_2_, and NOEs following standard equations (15, 35) using the Excel Solver Evolutionary method under the constraints that 0 ≤ S^2^ ≤ 1, 1 ns ≤ τ_m_ ≤ 100 ns, and τ_e_ ≤ τ_m_/9.

Three-bond H^N^-H^α^ J-coupling values were extracted from 3D HNHA spectra based on the intensities of the H^N^-H^N^ diagonal peak (I_diag_) and H^N^-H^α^ (I_cross_) crosspeaks using the equation ^3^J_HNHA_ = arctan[(−I_cross_/I_diag_)^1/2^]/(2πζ), where ζ = 13.05 ms (14). For HHQ and HHQ13, four ^3^J_HNHA_ values were measured, whereas for K7, ^3^J_HNHA_ values were measured for six residues. These couplings were specified with error bars of ±0.2 Hz. For the HHQ peptide, these ^3^J_HNHA_ couplings were fit using rigid-limit Karplus coefficients of (A, B, C) = (7.97, −1.26, 0.63) Hz as well as ensemble-averaged Karplus coefficients of (8.40, −1.36, 0.33) Hz (13). Both sets of parameters led to very similar ϕ torsion angles and did not affect the structure calculation.

### Xplor-NIH Structure Calculation.

The structures of HHQ, HHQ13, and K7 peptides were calculated based on measured ^1^H-^1^H NOEs and ^3^J_HNHA_ couplings using the Xplor-NIH software (36) hosted on NMRbox (37). The K7 structure calculation used data obtained in the first 3 d and thus represents the initial state of the peptide. A total of 1,000 independent simulated annealing runs were conducted, each starting with a random coil peptide. Each simulated annealing run was 200 ps long, with the temperature ramped from 5,000 K to 20 K in decrements of 10 K. At 5,000 K, high-temperature torsion angle dynamics were run for 100 ps or 5,000 steps, whichever was less. Lower temperatures used 100 steps or 0.2 ps of simulated annealing at each temperature. Standard bond angles, bond lengths, improper angles, repulsive nonbonded, and hydrogen bonding parameters were set using the potential terms BOND, ANGL, IMPR, RepelPot, and HBPot (*SI Appendix*, Table S3). We omitted the standard database dihedral potentials CDIH and torsionDB to avoid biasing the H_2_SO_4_ structural models toward aqueous protein geometry. Potential energy terms corresponding to the NOE restraints were ramped from 10 to 50 kcal/Å^2^, while terms corresponding to ^3^J_HNHA_ restraints were ramped from 2 to 30 kcal/Hz^2^. Hard-square and harmonic potential profiles were used for the NOE and J-coupling restraints, respectively.

Distance restraints from 2D ^1^H NOESY spectra were given lower bounds of 1.5 Å and upper bounds of 4.5 Å, 5.5 Å, 6.0 Å, and 6.5 Å for contacts first observed at 20 ms, 30 to 50 ms, 100 ms, and 300 ms, respectively (*SI Appendix*, Table S2). The upper bounds for mixing times of 20 ms and 30 to 50 ms were chosen to encompass the largest expected interproton distance among all resolved crosspeaks, based on calibration against a reference peak (Gln6 H^γ1^-H^γ2^ crosspeak in HHQ, Gln12 H^γ1^-H^γ2^ crosspeak in HHQ13 and Lys2 H^β1^-H^β2^ crosspeak in K7). For longer mixing times, the upper bound of distance restraint was moderately increased based on the previous mixing time. Distance restraints with ambiguous chemical shift assignments were inputted using an “or” statement, where each pair of atoms was evaluated during the calculation. For resolved crosspeaks between H^N^ of residue *i* and H^α^ of residue *i* or *i*-1, which are among the most intense peaks in the 20 ms NOESY spectrum, exact distances d_αN_(*i*, *i*) and d_αN_(*i*-1, *i*) were used for HHQ13 and K7. This was done by calibrating peak intensities relative to the Gln12 H^γ1^-H^γ2^ crosspeak in HHQ13 and Lys2 H^β1^-H^β2^ crosspeak in K7. These reference crosspeaks represent a distance of 1.8 Å based on standard covalent geometry of methylene groups. A total of 20 calibrated distances were specified for HHQ13 and 8 for K7. Errors for the d_αN_(*i*, *i*) and d_αN_(*i*, *i+1*) distance restraints were set to be ±10% of the calibrated distances. For HHQ, there was no difference in calculated structures with or without this calibration, indicating there are sufficient unambiguous restraints that define the fold. A total of 162, 277, and 145 NOE restraints were used for HHQ, HHQ13, and K7, respectively, for structure calculation (Table 1).

To verify whether the NOEs from the 300 ms NOESY spectra bias the structure due to potential relayed transfer, we conducted alternative structure calculation using NOESY spectra up to 100 ms mixing. These calculations resulted in the same omega-shaped conformation (*SI Appendix*, Fig. S5), only with slightly different curvature. We further validated model-predicted ^1^H-^1^H distances that are shorter than 5 Å between residues more than two apart in the sequence against observed crosspeaks in the spectra. For HHQ, 98 such distances are predicted in the lowest-energy structure, among which 28 are found in the spectra, 53 have crosspeaks that can be ambiguously assigned to these contacts. The remaining 17 contacts that are not found in the spectra involve exchangeable protons such as Gln6 H^ε^. For K7, 83 such distances are predicted in the lowest-energy structure, nine of which are unambiguously assigned in the spectra and 74 of which are ambiguously assigned. For HHQ13, 271 distances are predicted by the structure model, 17 of which are unambiguously assigned in the spectra and 235 are ambiguously assigned. The remaining 19 contacts are not found in the spectra, but all involve exchangeable or unassigned side chain proton resonances.

### Molecular Docking.

The crystal structure of H_2_SO_4_ (38) was downloaded from the Crystallography Open Database (COD #1535480). The HADDOCK software (39) was used to dock H_2_SO_4_ onto the HHQ, HHQ13, and K7 structures. To identify the active residues, the H^N^-solvent crosspeak intensities were compared to the 1D H^N^ peak intensities and normalized to the highest observed ratio. Residues with a normalized intensity ratio of at least 50% were selected as “active” in HADDOCK simulations. The active residues were Val3, Gln6, and Ile7 for HHQ, Ala3, Ala7, and Gln12 for HHQ13, and Lys2, Lys5, Ala6, and Val7 for K7. Other residues of the peptides were defined as passive and semi-rigid. Distance restraints for backbone H^N^ to H_2_SO_4_ oxygen atoms were specified with an upper bound of 2.0 Å for active residues and an upper bound of 2.5 Å for passive residues. The protonation state of all histidines were set to positively charged His^+^, and all residues were allowed to be semiflexible. Docking simulations included 1,000 runs for rigid-body docking, 200 runs for semiflexible refinement and 200 runs for final refinement, with clustering based on the ligand interface rmsd using a cutoff of 7.5 Å. The other parameters were set to default values. Binding energies in kcal/mol for the best docking sites were calculated using PRODIGY-LIGAND, a web server for predicting binding affinity in protein-small ligand complexes (40).

### Peptide Selection and Design.

To design peptides that may adopt a stable fold in concentrated sulfuric acid, we assumed a minimum peptide length and excluded the amino acid that is reactive in concentrated sulfuric acid (tryptophan) and amino acids that are readily modified on their sidechains by sulfation (methionine, serine, threonine, or cysteine) or sulfonation (phenylalanine or tyrosine) (7). For ease of structure determination, we avoided palindromic sequences and minimized repeated amino acids.

## Supplementary Material

Appendix 01 (PDF)

## Data Availability

The peptide structures have been deposited into the Protein Data Bank (PDB) and the NMR data have been deposited into Biological Magnetic Resonance Bank (BMRB). The PDB and BMRB codes are 12UF (41) and 31302 (42) for HHQ, 12UG (43) and 31303 (44) for HHQ13, and 12UI (45) and 31304 (46) for K7.
