# Supplementary material for: Peptides adopt stable omega-loop structures in concentrated sulfuric acid
Source: Proc Natl Acad Sci U S A. 2026 Sep 4;123(37):e2618039123. doi: 10.1073/pnas.2618039123 (PMC13578377; doi:10.1073/pnas.2618039123)
Supplement: Supplementary file 1 [file pnas.2618039123.sapp.pdf]

PNAS

Supporting Information for

Peptides Adopt Stable Omega Loop Structures in Concentrated Sulfuric Acid

Jia Yi Zhang [1], Aurelio J. Dregni [1], Janusz J. Petkowski [2,3*], Sara Seager [4,5,6*] and Mei Hong [1*]

[1] Department of Chemistry, Massachusetts Institute of Technology, 170 Albany Street, Cambridge, MA 02139, United States

[2] Faculty of Environmental Engineering, Wroclaw University of Science and Technology, 50-370, Wroclaw, Poland

[3] JJ Scientific, Mazowieckie, Warsaw 02-792, Poland

[4] Department of Earth, Atmospheric and Planetary Sciences, Massachusetts Institute of Technology, 77 Massachusetts Avenue, Cambridge, MA 02139, United States

[5] Department of Physics, Massachusetts Institute of Technology, Cambridge, MA 02139, United States

[6] Department of Aeronautics and Astronautics, Massachusetts Institute of Technology, Cambridge, MA 02139, United States

* Corresponding author: Mei Hong, Sara Seager, Janusz J. Petkowski

Email: meihong@mit.edu, seager@mit.edu, janusz.petkowski@pwr.edu.pl

This PDF file includes:

Figures S1 to S11
Tables S1 to S5

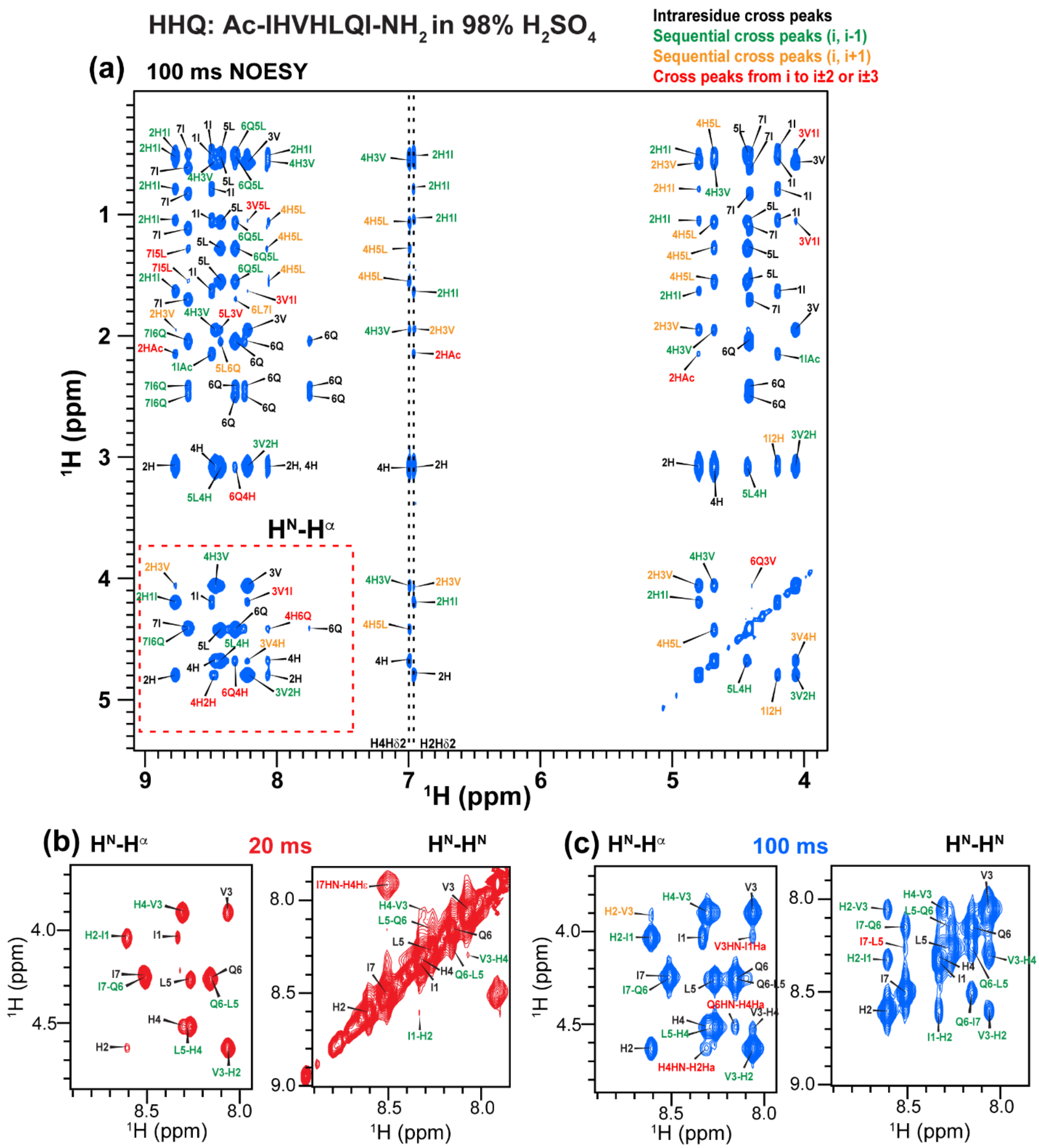

Fig. S1. **(a)** Resonance assignment of aliphatic region of the 100 ms 2D ^{1}H NOESY spectrum of HHQ in 98% H_2SO_4. Assignment is annotated in the order of (ω_2, ω_1). Intra-residue assignments are shown in black, sequential (*i*, *i-1*) crosspeaks in green, sequential (*i*, *i*+1) crosspeaks in orange, and longer-range crosspeaks in red. (**b**) H^N-H^α and H^N-H^N regions of the 20 and 100 ms NOESY spectra, showing stronger sequential αN cross peaks than sequential NN cross peaks, indicating that the crosspeak intensities reflect inter-proton distances and are not simply a consequence of solvent-peptide exchange.

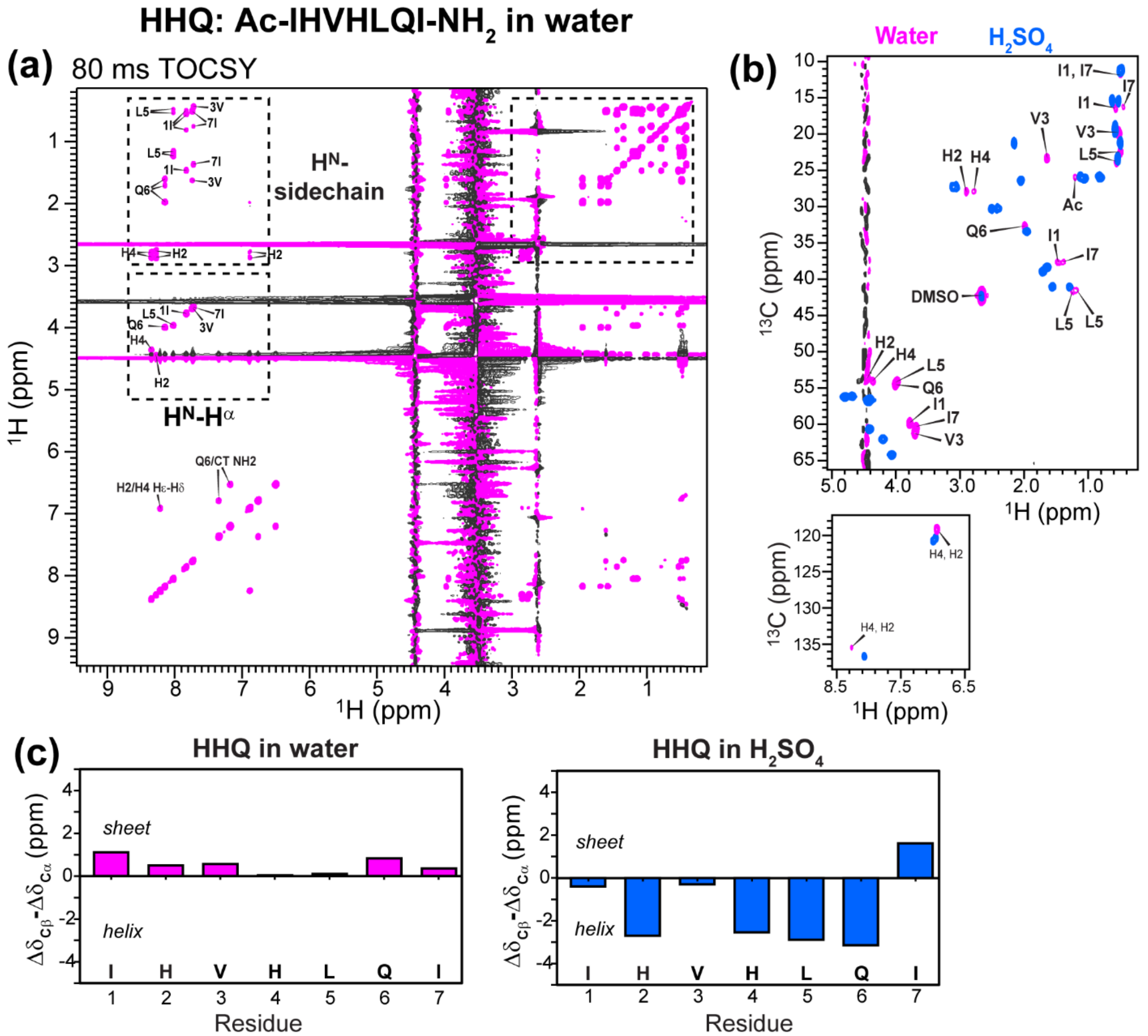

Fig. S2. HHQ spectra in water. **(a)** 2D ^{1}H TOCSY spectrum. Positive intensities are shown in magenta and negative intensities are shown in gray. **(b)** Aliphatic (top) and aromatic (bottom) regions of the 2D ^{13}C-^{1}H HMQC spectra of HHQ in water (magenta) versus in 98% H_2SO_4 (blue). **(c)** Cα and Cβ secondary chemical shifts of HHQ in water versus in 98% H_2SO_4.

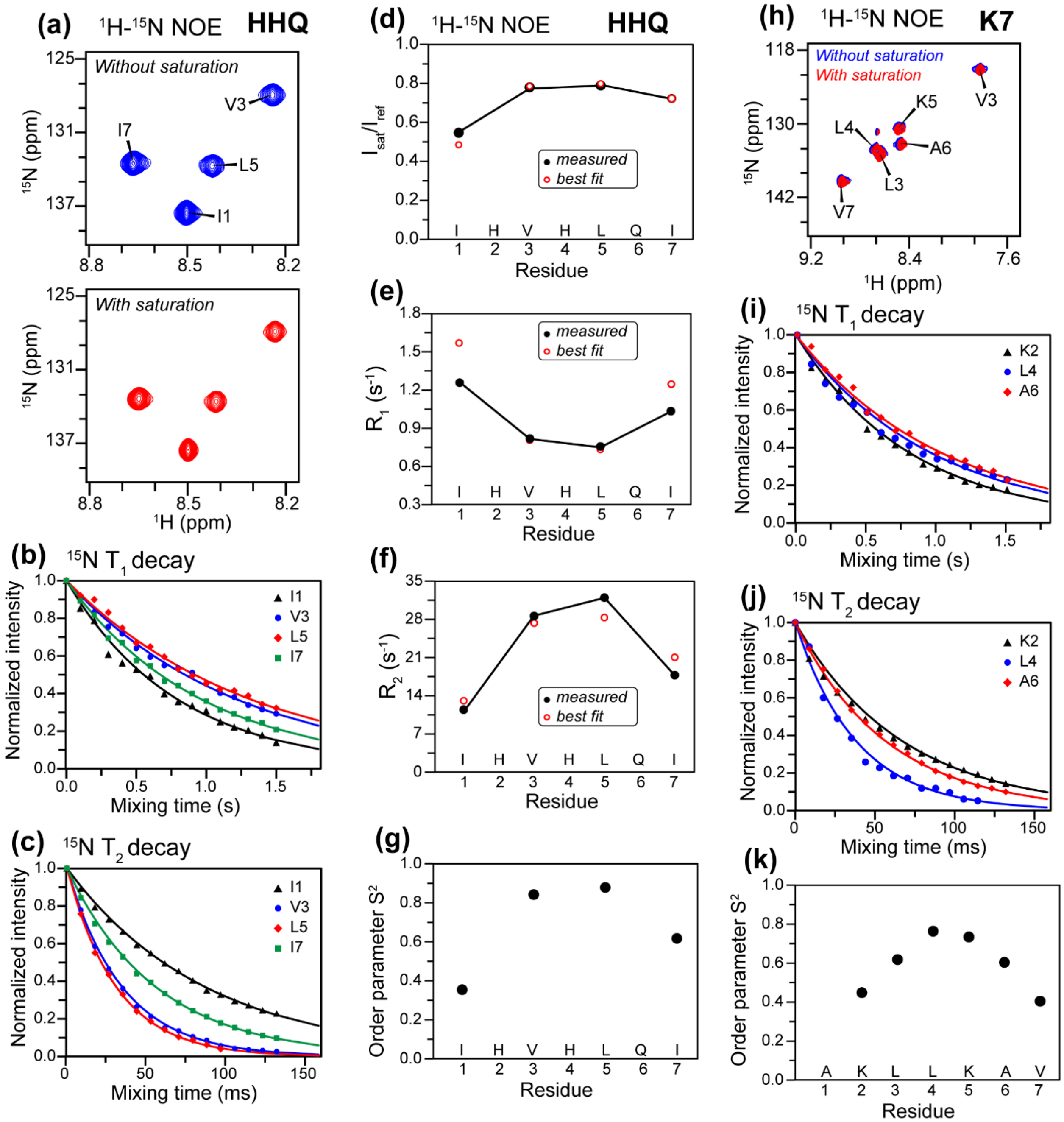

Fig. S3. ^{15}N relaxation NMR data of HHQ (**a-g**) and K7 (**h-k**) peptides in 98% sulfuric acid. (**a**) 2D ^{1}H-^{15}N HSQC spectra measured without and with ^{1}H presaturation for 5 s. (**b**) ^{15}N T_1 relaxation decays of HHQ for the four ^{15}N-labeled residues. (**c**) ^{15}N T_2 relaxation decays. (**d**) Steady-state ^{1}H-^{15}N NOE values extracted from the spectra in (**a**). (**e**) Measured ^{15}N longitudinal relaxation rates R_1. (**f**) Measured ^{15}N R_2 relaxation rates. (**g**) Best-fit N-H order parameter S^2 obtained from the R_1, R_2 and NOEs. In (**d-f**), best-fit values (red circles) are compared with measured values (black circles). (**h**) 2D HSQC spectra of K7 without and with ^{1}H presaturation. (**i**) ^{15}N T_1 relaxation decays for selected residues. (**j**) ^{15}N T_2 relaxation decays. (**k**) Best-fit S^2 values obtained from the measured ^{15}N R_1, R_2 and heteronuclear NOEs.

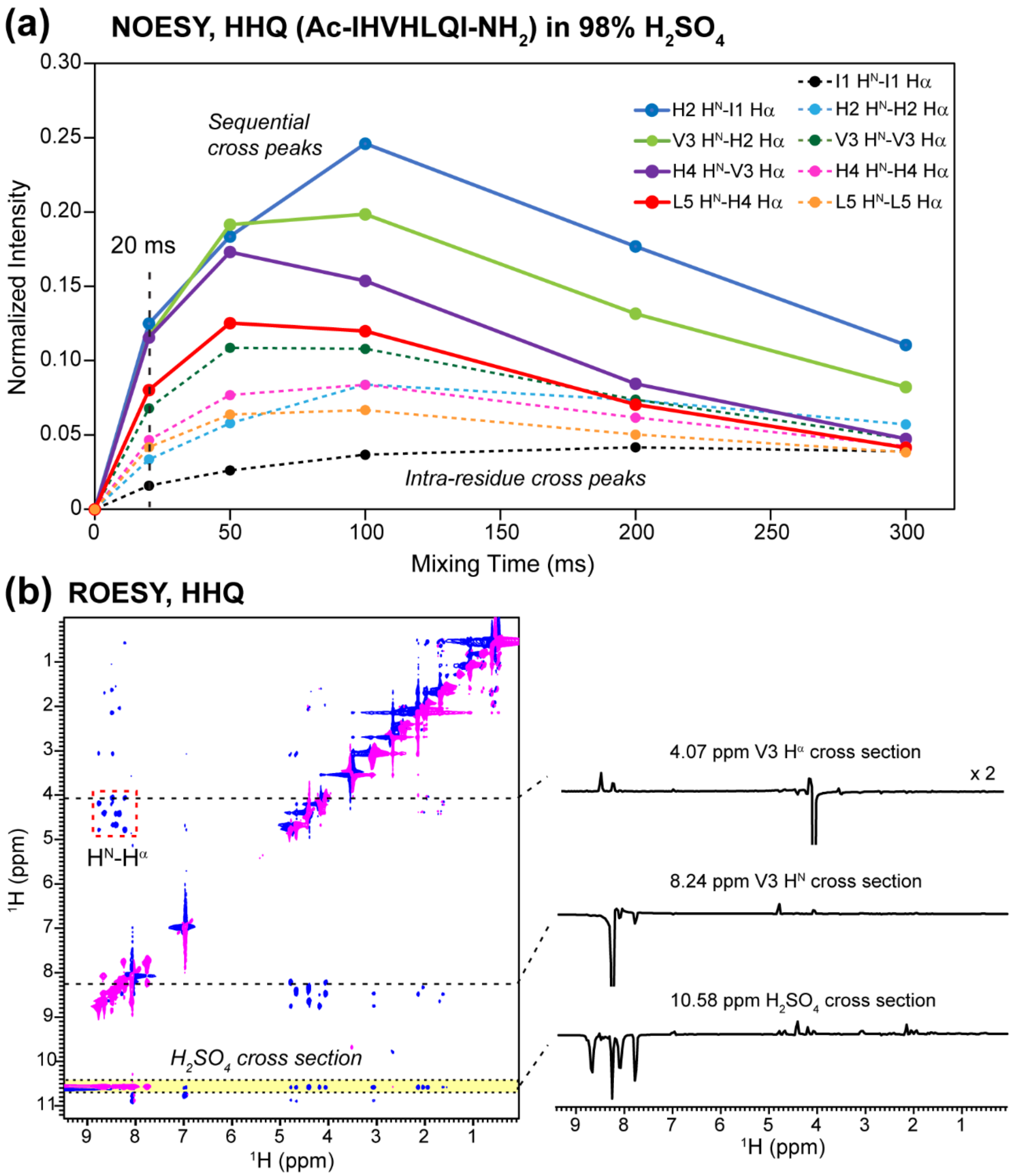

Fig. S4. ^{1}H NOESY buildup and ROESY data of HHQ in 98% H_2SO_4. (**a**) NOE buildup curves of sequential (solid lines) and intra-residue (dashed lines) H^α-H^N crosspeaks, obtained from spectra measured with 20, 50, 100, 200 and 300 ms mixing. Crosspeak intensities were normalized to the extrapolated 0 ms spectrum's diagonal intensities, which were obtained by fitting the diagonal peak intensities across the mixing times to an exponential function. Even at the shortest mixing time of 20 ms, sequential H^α-H^N crosspeaks are stronger than intra-residue H^α-H^N crosspeaks. The linear and sub-linear buildup curves indicate that these intra-residue and sequential H^α-H^N cross peaks result from direct contacts. (**b**) 10 ms ^{1}H ROESY spectrum of HHQ, measured with a 5 kHz spin-lock field on-resonance with the solvent peak at 10.58 ppm. Positive intensities are shown in blue and negative intensities in pink. The solvent-peptide cross peaks have the same sign as diagonal peaks, indicating that chemical exchange dominates the peptide-solvent magnetization transfer. The peptide H^α-H^N crosspeaks have opposite signs from the diagonal, consistent with direct dipolar magnetization transfer.

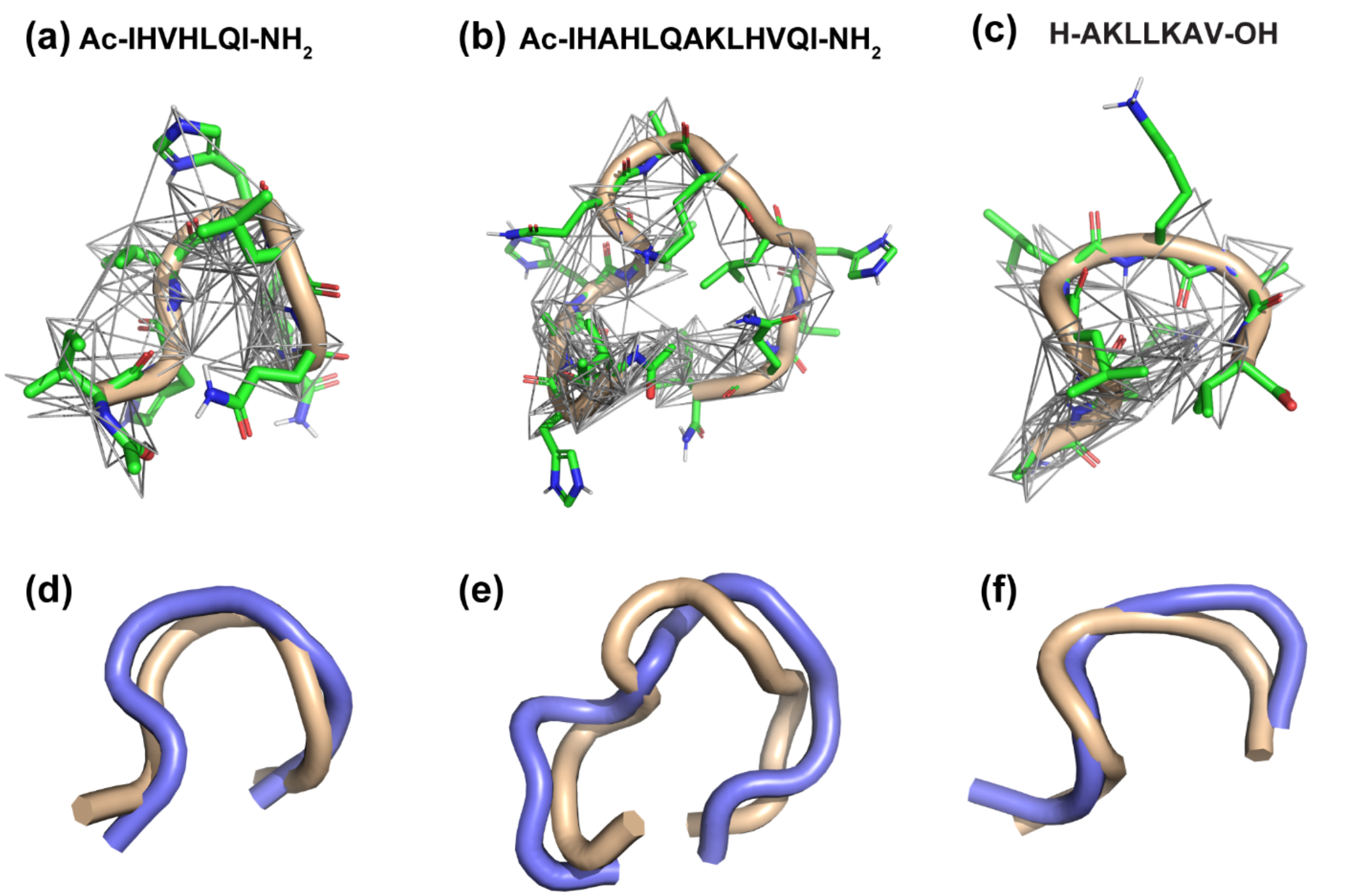

Fig. S5. Structure calculations of the three peptides in 98% H_2SO_4. (**a-c**) Structure models calculated using all measured NOEs. (**a**) Lowest-energy structure model of HHQ, showing 162 NOE restraints (gray lines). **(b)** Lowest-energy structure model of HHQ13, restrained by 237 NOEs. Fewer NOEs are available for K8 and L9 due to spectral overlap and ambiguous resonance assignment. Thus, these two residues are less constrained, whereas the first 7 residues of the peptide are well constrained with a heavy-atom RMSD of 0.4 Å. **(c)** Lowest-energy structure model of K7, restrained by 114 NOEs. (**d-f**) Backbone structural models calculated without 300 ms NOEs (blue cartoons), overlaid with models calculated with all NOEs (wheat cartoons). The same omega-loop conformations are found, except with slightly different curvatures. Thus, the omega-loop fold is insensitive to the exact assumptions in structure calculation. Heavy-atom RMSDs for the lowest-energy ensembles without the 300 ms NOEs are (**d**) 1.25 Å for HHQ, (**e**) 1.98 Å for HHQ13, and (**f**) 1.69 Å for K7. Between the lowest-energy structural models calculated with and without the 300 ms NOEs, backbone RMSDs are 1.6 Å for HHQ, 3.0 Å for HHQ13, and 1.4 Å for K7.

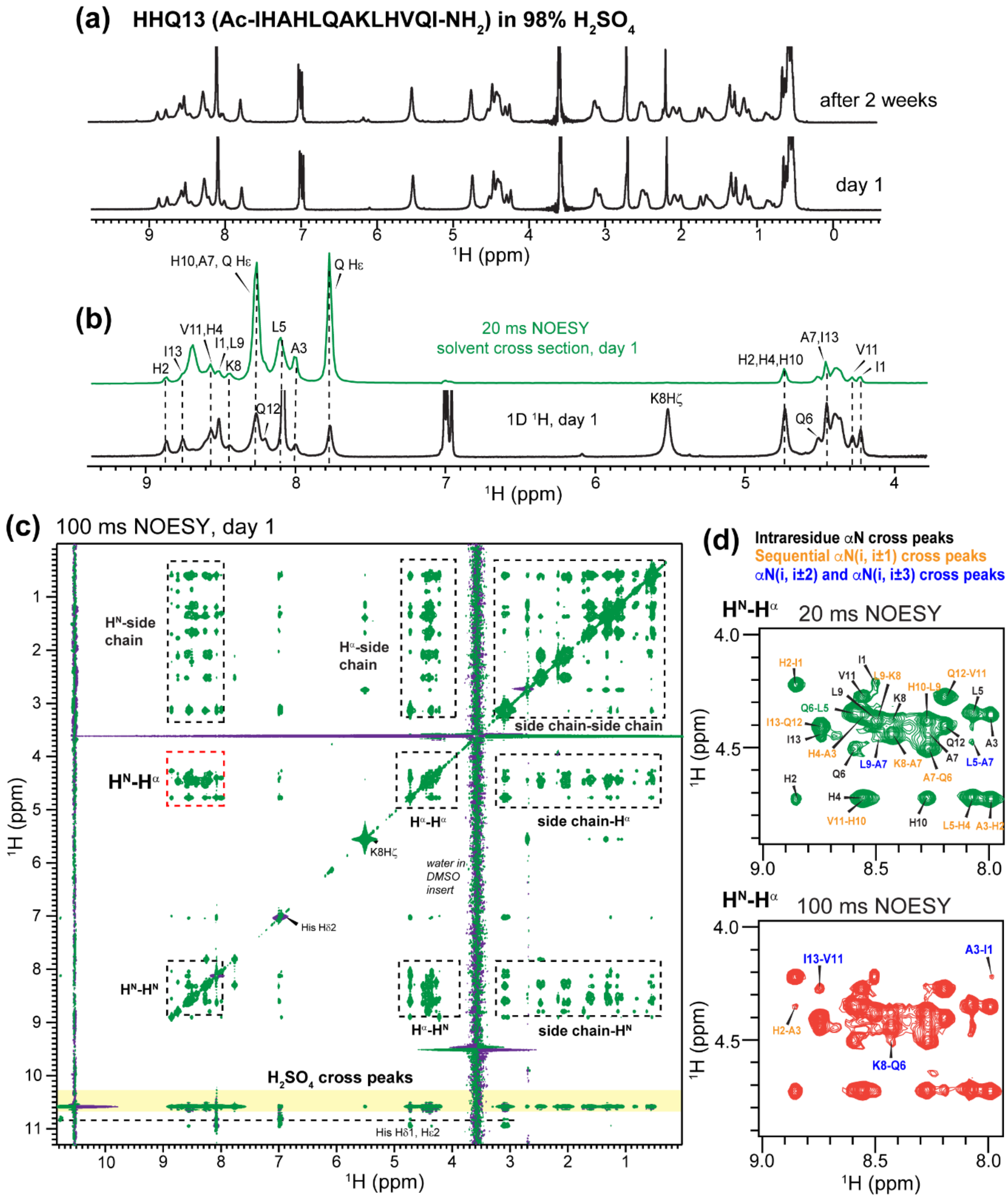

Fig. S6. 1D and 2D ^{1}H NMR spectra of HHQ13 in 98% H_2SO_4. **(a)** ^{1}H spectra of the peptide on day 1 and after 2 weeks of being in 98% H_2SO_4 at room temperature. The spectra show little change, indicating the peptide is stable. **(b)** Solvent cross section extracted from the 20 ms NOESY spectrum compared to the 1D ^{1}H spectrum. Different H^N intensity patterns indicate different proximities of the H^N protons from solvent molecules. **(c)** 100 ms 2D ^{1}H NOESY spectrum of HHQ13 on day 1. **(d)** H^N-H^α fingerprint region of the 20 ms and 100 ms NOESY spectra. Crosspeak assignment is color-coded in black for intra-residue peaks, orange for sequential peaks, and blue for other peaks. In the 100 ms spectrum, only peaks that are absent in the 20 ms spectrum are assigned.

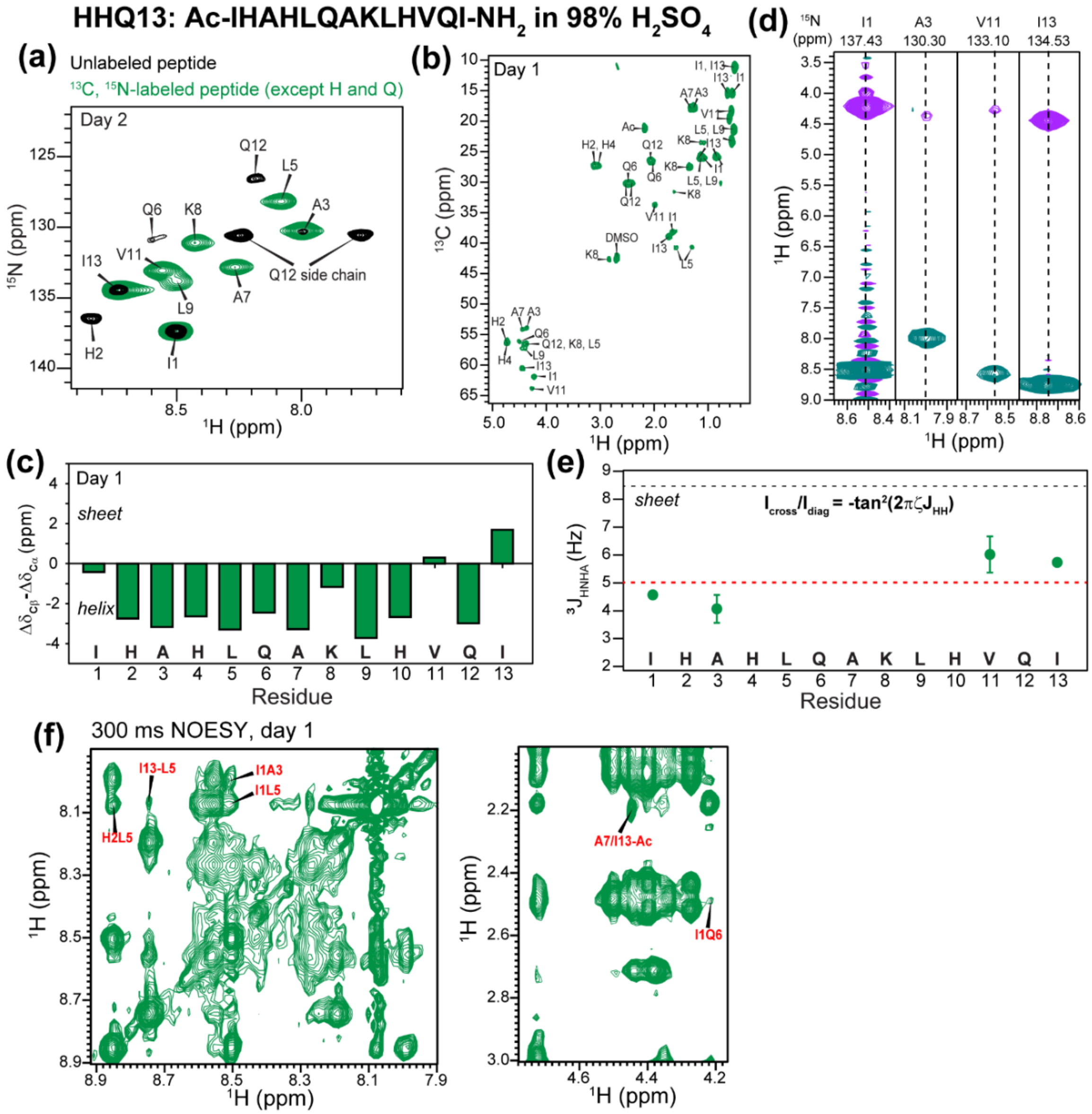

Fig. S7. 2D and 3D heteronuclear correlation spectra of HHQ13 in 98% H_2SO_4. **(a)** 2D ^{1}H-^{15}N HSQC spectrum. Only His and Gln peaks are observed in the natural abundance spectrum (black), while residues in the middle of the peptide show strong signals only in the ^{13}C, ^{15}N-labeled sample (green). **(b)** Aliphatic region of the 2D ^{1}H-^{13}C HMQC spectrum. **(c)** Cα and Cβ secondary chemical shifts. **(d)** 3D J_{HNHA} spectral strips. Positive H^N diagonal peaks are shown in green and negative H^N-H^α crosspeaks are shown in purple. **(e)** $^3J_{HNHA}$ couplings of labeled residues of HHQ13. The couplings average at ~5 Hz (red dashed line), indicating that the peptide does not adopt an extended structure. **(f)** Regions of the 300 ms NOESY spectrum, showing several long-range contacts, including Ile1-Leu5, Ile1-Gln6, and Ile13-Leu5, which constrain the peptide structure.

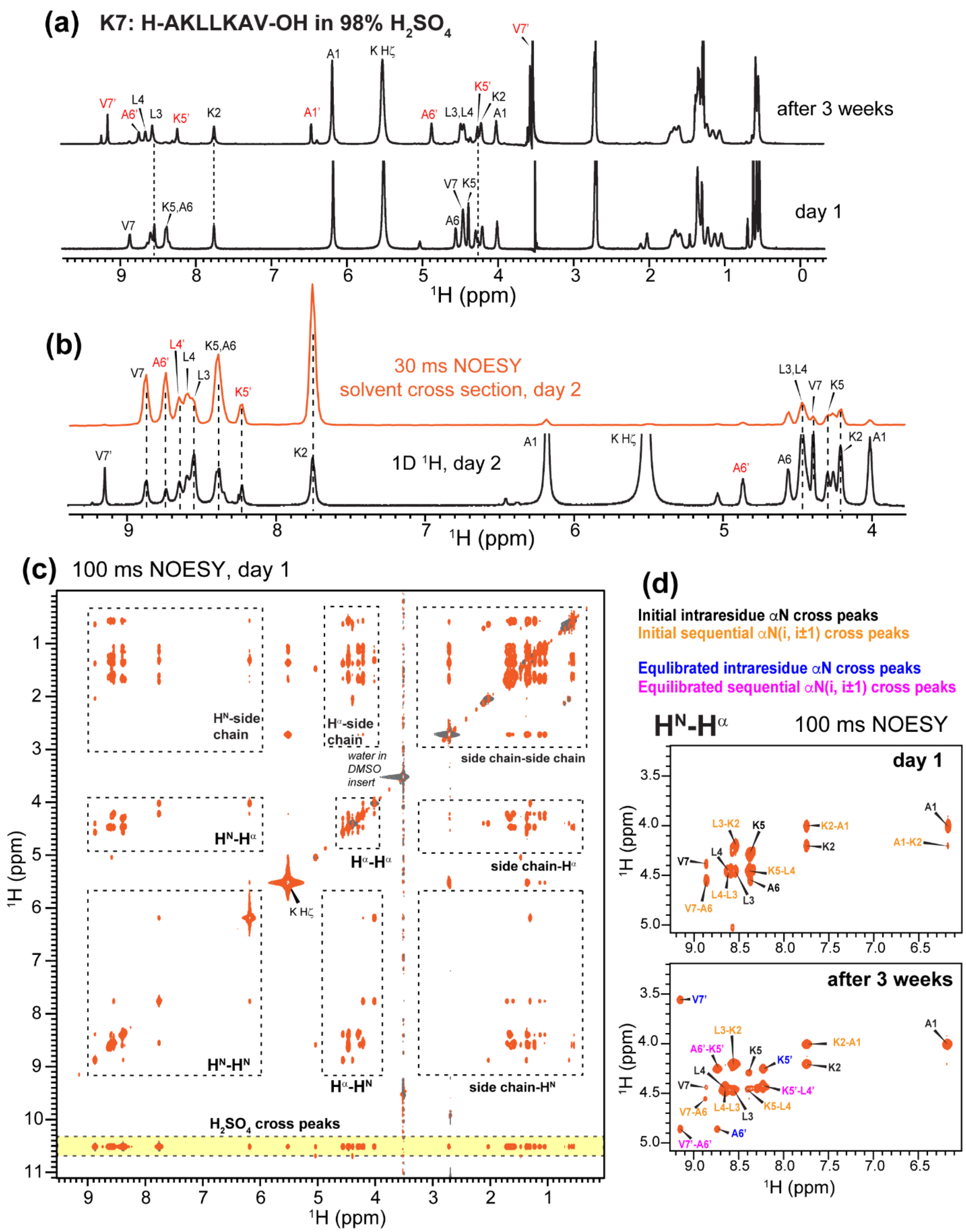

Fig. S8. 1D and 2D ^1H NOESY spectra of K7 in 98% H_2SO_4 over three weeks at room temperature. (**a**) ^1H spectra on day 1 and after 3 weeks. Chemical shift changes indicate that the peptide structure slowly changed in three weeks. (**b**) Solvent cross section of the peptide from the 30 ms 2D NOESY spectrum on day 2, showing a similar intensity pattern as the 1D ^1H spectrum. (**c**) 100 ms 2D NOESY spectrum of the peptide on day 1. Strong solvent crosspeaks were observed. (**d**) H^N-H^α fingerprint region of the NOESY spectra on day 1 and after 3 weeks. For the initial state, intra-residue peaks are assigned in black while sequential crosspeaks are assigned in orange. For the equilibrated state, intra-residue peaks are assigned in blue and sequential crosspeaks are assigned in magenta.

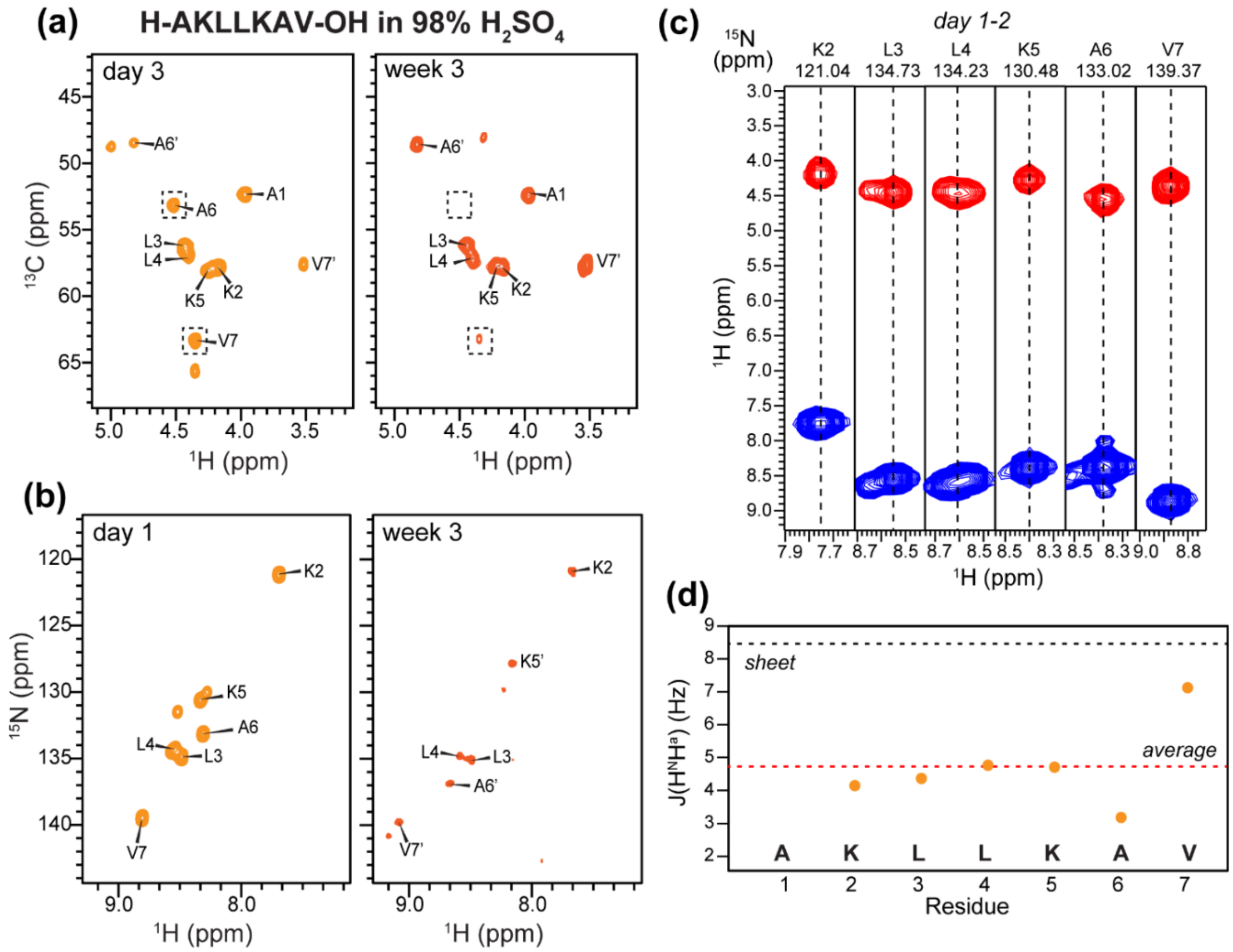

Fig. S9. 2D and 3D correlation spectra of K7 in 98% H_2SO_4 at room temperature. **(a)** 2D ^{13}C-^{1}H HMQC spectra on day 3 and in week 3. Primed assignments indicate a second conformation. **(b)** 2D ^{15}N-^{1}H HSQC spectra on day 1 and in week 3. **(c)** 3D HNHA spectrum in the initial state. The relative intensities of the positive H^N diagonal peaks (blue) and negative H^N-H^α crosspeaks (red) give the $^3J_{HNHA}$ value. **(d)** $^3J_{HNHA}$ couplings of the initial state of K7 average around 4.7 Hz. Error bars are 0.05 Hz and are not shown.

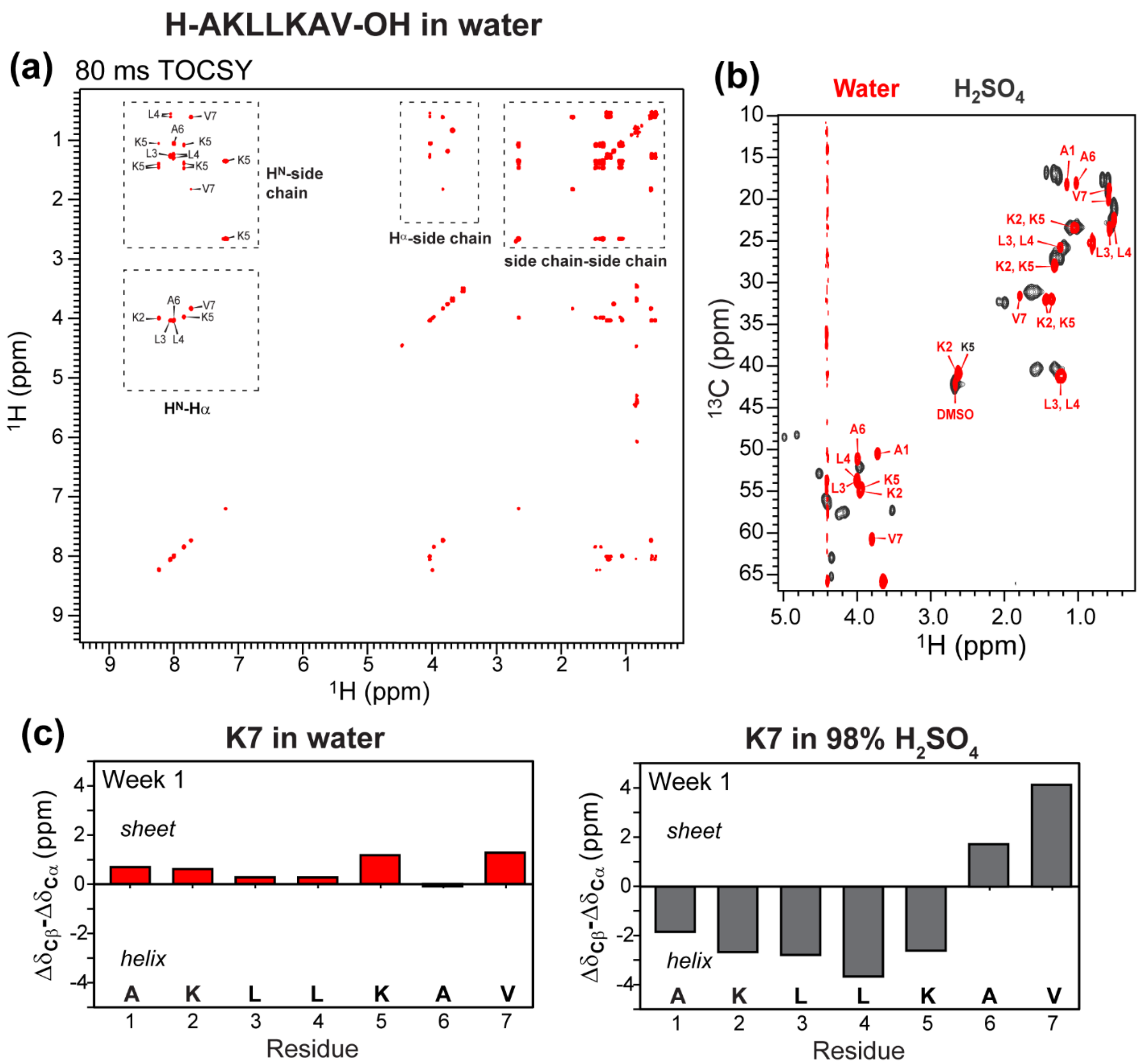

Fig. S10. 2D NMR spectra of K7 in water at room temperature. (**a**) 2D ^1H-^1H TOCSY spectrum measured with 80 ms mixing. (**b**) 2D ^{13}C-^1H HMQC spectra in water (red) versus in H_2SO_4 (gray). (**c**) Cα and Cβ secondary chemical shifts of K7 in water versus in 98% H_2SO_4. K7 has distinct conformational propensities in the two solvents: it is random coil with a weakly β-strand propensity in water but has helical propensity in 98% H_2SO_4.

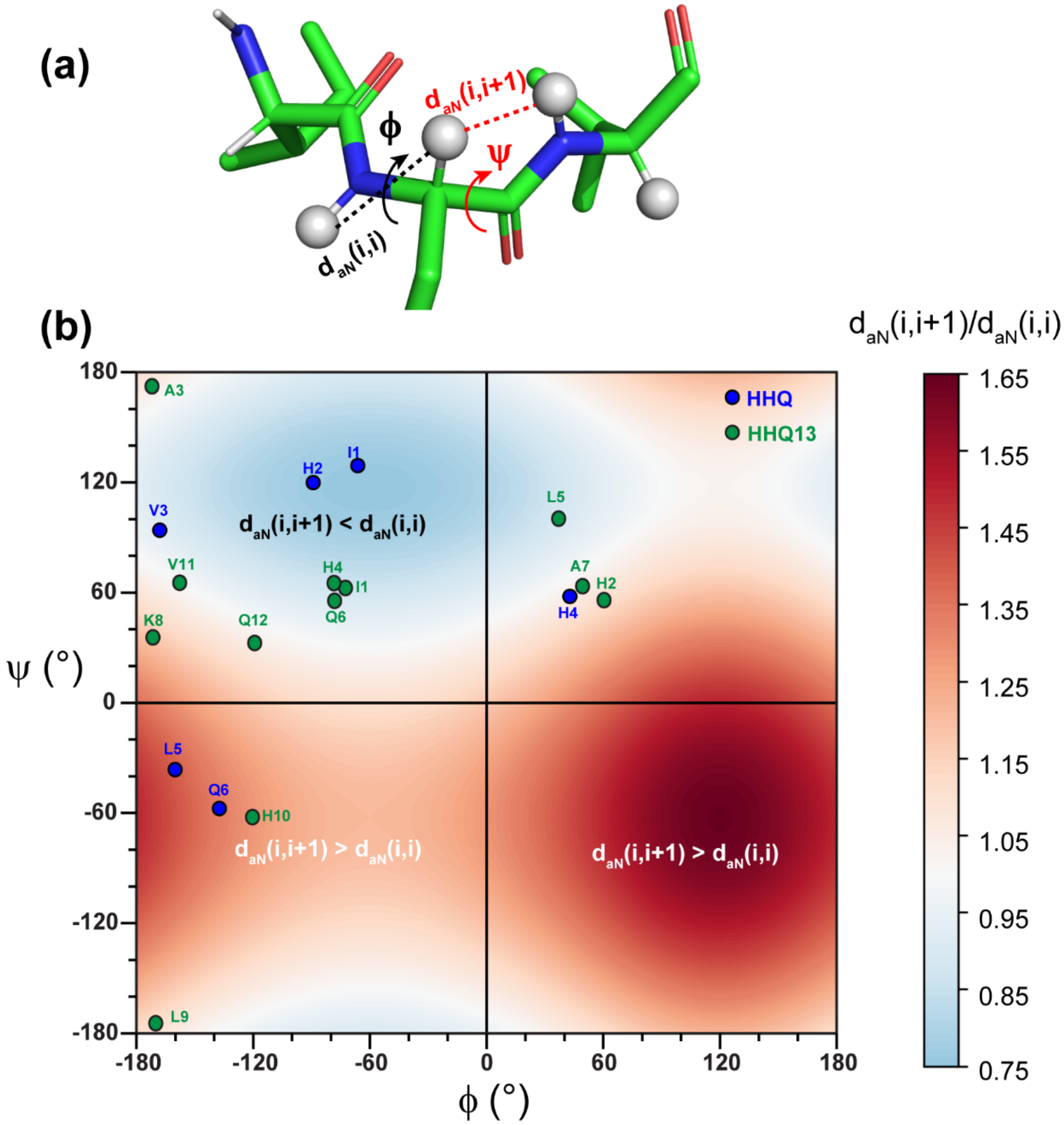

Fig. S11. (ϕ, ψ) torsion angles adopted by omega-loop peptides in concentrated sulfuric acid. **(a)** Representative view of a peptide backbone. The αN(*i*, *i*) distance depends on the ϕ torsion angle of residue *i* whereas the αN(*i*, *i*+1) distance depends on the ψ angle of residue *i*. **(b)** Contour of the ratio of αN(*i*, *i*+1) distance to αN(*i*, *i*) distance as a function of (ϕ, ψ) torsion angles. Shorter sequential αN(*i*, *i*+1) distances than intra-residue αN(*i*, *i*) distances are found in the upper left quadrant of the Ramachandran diagram (blue shades). The torsion angles in the lowest-energy structures of HHQ and HHQ13 are distributed in this region (circles), where most residues adopt positive ψ angles and either positive or negative ϕ angles.

Table S1. ^{15}N relaxation data and best-fit S^2 and internal correlation times τ_e for HHQ in 98% sulfuric acid. The rotational correlation time is 19.3 ns.

Residue	^{15}N R_1 (s^{-1})	^{15}N R_2 (s^{-1})	^{15}N NOE	S^2	τ_e (ns)
1	1.26	11.43	0.55	0.36	1.1
3	0.82	28.58	0.78	0.84	2.0
5	0.76	31.95	0.79	0.88	2.0
7	1.04	17.77	0.72	0.62	1.8

Table S2. Number of unambiguously assigned NOEs at various mixing times for the three peptides in 98% H_2SO_4. Only new NOEs absent in shorter mixing time spectra are listed.

Contact type	20 ms		30 ms	50 ms	100 ms			300 ms		
	HHQ	HHQ13	K7	HHQ	HHQ	HHQ13	K7	HHQ	HHQ13	K7
Intra-residue	60	90	58	0	0	16	0	0	1	2
(i, i±1)	27	44	36	15	11	43	0	14	6	3
(i, i±2)	0	2	5	1	6	12	1	20	14	3
(i, i±3)	0	0	0	0	1	2	1	6	3	3
Long-range	1	1	1	0	0	0	0	0	3	1
Total	88	137	100	16	18	73	2	40	27	12
Upper bound distance	4.5 Å		5.5 Å	5.5 Å	6.0 Å			6.5 Å		

Table S3. XPLOR-NIH parameters and energies of HHQ, HHQ13, and K7 structures in 98% H_2SO_4.

			Best model energies (kcal/mol)		
**XPLOR-NIH potential**	**Experimental Basis**	**Scale Factor**	**HHQ**	**HHQ13**	**K7**
NOE	^1H-^1H NOESY crosspeaks	2-30	14.37	16.43	38.51
J-couplings	HNHA	2-30	0.28	0.05	0.20
BOND	Standard bond lengths	1	5.39	5.16	6.77
ANGL	Standard bond angles	0.4-1	15.18	19.83	22.97
IMPR	Standard bond geometry	0.1-1	6.42	2.09	2.84
RepelPot	Non-bonded atomic radii repulsion	0.004-4	19.54	20.48	11.67
HBPot	H-bonding	2.6	-3.99	-0.01	-0.10

Table S4. Input for H_2SO_4 docking simulations and binding energies of the docked complex calculated using PRODIGY-LIGAND.

Peptide	Active residues	# NOE restraints	ΔG (kcal/mol)
HHQ	Val3, Gln6, Ile7	9	-8.77
HHQ13	Ala3, Ala7, Gln12	14	-9.50
K7	Lys2, Lys5, Ala6, Val7	7	-9.05

Table S5. ^{15}N relaxation data and best-fit order parameters and internal correlation times for K7 in 98% sulfuric acid. The rotational correlation time is 19.3 ns.

Residue	^{15}N R_1 (s^{-1})	^{15}N R_2 (s^{-1})	^{15}N NOE	S^2	τ_e (ns)
2	1.21	14.9	0.63	0.45	1.4
3	1.12	18.2	0.77	0.62	1.6
4	1.02	26.1	0.81	0.76	2.2
5	0.92	18.2	0.76	0.73	1.4
6	0.95	17.6	0.82	0.60	2.1
7	1.27	12.3	0.66	0.40	1.5
